# Epigenetic clocks and their association with trajectories in perceived discrimination and depressive symptoms among US middle-aged and older adults

**DOI:** 10.18632/aging.204150

**Published:** 2022-07-01

**Authors:** May A. Beydoun, Hind A. Beydoun, Nicole Noren Hooten, Ana I. Maldonado, Jordan Weiss, Michele K. Evans, Alan B. Zonderman

**Affiliations:** 1 Laboratory of Epidemiology and Population Sciences, NIA/NIH/IRP, Baltimore, MD 21224, USA; 2 Department of Research Programs, Fort Belvoir Community Hospital, Fort Belvoir, VA 22060, USA; 3 Department of Psychology, University of Maryland, Baltimore County, Catonsville, MD 21250, USA; 4 Department of Demography, University of California Berkeley, Berkeley, CA 94720, USA

**Keywords:** DNA methylation, epigenetic clocks, biological age, perceived discrimination, depressive symptoms

## Abstract

Background: Perceived discrimination may be associated with accelerated aging later in life, with depressive symptoms acting as potential mediator.

Methods: A nationally representative sample of older adults was used [Health and Retirement Study 2010–2016, Age: 50–100 y in 2016, *N* = 2,806, 55.6% female, 82.3% Non-Hispanic White (NHW)] to evaluate associations of perceived discrimination measures [Experience of discrimination or EOD; and Reasons for Perceived discrimination or RPD) and depressive symptoms (DEP)] with 13 DNAm-based measures of epigenetic aging. Group-based trajectory and four-way mediation analyses were used.

Results: Overall, and mostly among female and NHW participants, greater RPD in 2010–2012 had a significant adverse total effect on epigenetic aging [2016: DNAm GrimAge, DunedinPoAm38 (MPOA), Levine (PhenoAge) and Horvath 2], with 20–50% of this effect being explained by a pure indirect effect through DEP in 2014–2016. Among females, sustained elevated DEP (2010–2016) was associated with greater LIN DNAm age (β ± SE: +1.506 ± 0.559, *p* = 0.009, reduced model), patterns observed for elevated DEP (high vs. low) for GrimAge and MPOA DNAm markers. Overall and in White adults, the relationship of the Levine clock with perceived discrimination in general (both EOD and RPD) was mediated through elevated DEP.

Conclusions: Sustained elevations in DEP and RPD were associated with select biological aging measures, consistently among women and White adults, with DEP acting as mediator in several RPD-EPICLOCK associations.

## INTRODUCTION

Epigenetics impacts gene expression, genome integrity and normal cell function [1, 2] through heritable changes that are independent of DNA sequence modifications such as mutations. DNA methylation (DNAm) is the most understood epigenetic mechanism [2] and occurs through addition of a methyl group to a CpG site in DNA [3]. Hypermethylation generally triggers gene expression silencing, while the reverse is true for hypomethylation [2]. DNA Methylation (DNAm) is also a mechanism by which exposure to adverse life circumstances and environments is linked to health outcomes related to aging [3]. It has been linked to psychopathology, including post-traumatic stress [4], major depressive disorder (MDD) and elevated depressive symptoms [5, 6] as well as cognitive aging [7, 8].

“Epigenetic clocks” derived from DNAm are mathematical models reflecting human cell, tissue, and organ aging, while being highly correlated with age across the life span [9] and to increased age-related chronic disease and all-cause mortality risk [10]. These clocks combine information for a small number of CpGs (~100–500) to produce indicators of aging [3]. Methylation clocks are estimated in epigenetic years with the rationale that ticks of the clock represent aging [3].

The Horvath and Hannum “epigenetic clocks” are well-established epigenetic age algorithms whereby DNAm can be utilized to estimate biological aging at the cellular level [11]. Since then, a number of other researchers have identified epigenetic clocks based on different genomic methylation changes that are related to age or health outcomes linked to age [3]. Despite differences in these algorithms and loci, both the Horvath and Hannum approaches, for instance, produce clocks that are strongly associated with chronological age [11]. Generally, epigenetic age acceleration or faster “epigenetic clock” has been linked to health decline including higher mortality risk [12] and faster cognitive decline [7, 9, 13, 14]. However, few epidemiological studies have directly linked epigenetic clocks or DNAm to MDD [15–17] and only two have directly or indirectly examined its association with elevated depressive symptoms [5, 6].

Antecedent psychosocial factors to depressive symptoms may be at play in explaining racial/ethnic and gender disparities in biological aging [18]. Among these psychosocial factors, perceived discrimination has been linked to adverse health outcomes, possibly through stress-related pathways involving hypertension, cardiovascular disease, poor general health status, and mental illness [18, 19]. Stress is a condition whereby environmental factors tax or exceed the adaptive capacity of individuals to a point where psychological and physiological responses may place them at risk for disease [20]. Studies of stressors and their relation to pathophysiology have revealed alterations in blood pressure, heart rate and vascular reactivity in response to acute stress [21], which may be mediated through measures of biological aging. We hypothesize that sustained perceived discrimination and depressive symptoms over time are associated with accelerated aging later in life. We also hypothesize that elevated depressive symptoms may mediate the association between perceived discrimination and biological aging as determined by DNAm epigenetic clocks. Differences in depressive symptoms by sex and race have also been detected [22]. Thus, it is important to uncover the relationship between epigenetic aging, perceived discrimination and depressive symptoms while stratifying by sex and race/ethnicity.

We used data from the nationally representative and longitudinal Health and Retirement Study (HRS) to examine the extent to which measures of perceived discrimination and depression were associated with epigenetic aging of HRS respondents. We further examined mediation/moderation hypotheses between perceived discrimination and depression as well as how these associations may vary by sex and race/ethnicity.

## MATERIALS AND METHODS

### Database

The HRS is an ongoing, nationally representative longitudinal study of community-dwelling U.S. adults over the age of 50 and their spouses of any age with interviews occurring every two years since 1992. The HRS was designed as a study of economic well-being, labor force participation, health and family composition among older adults through biennial surveys administered by telephone or face-to-face interviews. Even though the HRS interviews were initially conducted only on community-dwelling adults, respondents who transition into long-term care facilities are also retained. The sampling strategy of HRS is a multistage probability selection of U.S. households within geographical strata, whereby African Americans, Hispanics and residents of Florida were over-sampled. Baseline and follow-up response rates were >80% for all HRS interviews. All participants provided written informed consent and the University of Michigan’s Institutional Review Board approved study protocols. An important scientific goal was to combine HRS with the AHEAD study into a single ongoing survey that would be continually representative of the complete U.S. population over the age of 50. Thus, new birth cohorts were added to achieve this goal over the years to a achieve steady state design. In 2016, a subsample of 4,104 participants in the Health and Retirement 2016 Venous Blood Study (VBS) consented to providing biological samples upon which DNA methylation assays were conducted [3]. The HRS is sponsored by the National Institute on Aging (grant number U01AG009740) and the Social Security Administration.

### Study sample

**Figure 1:**
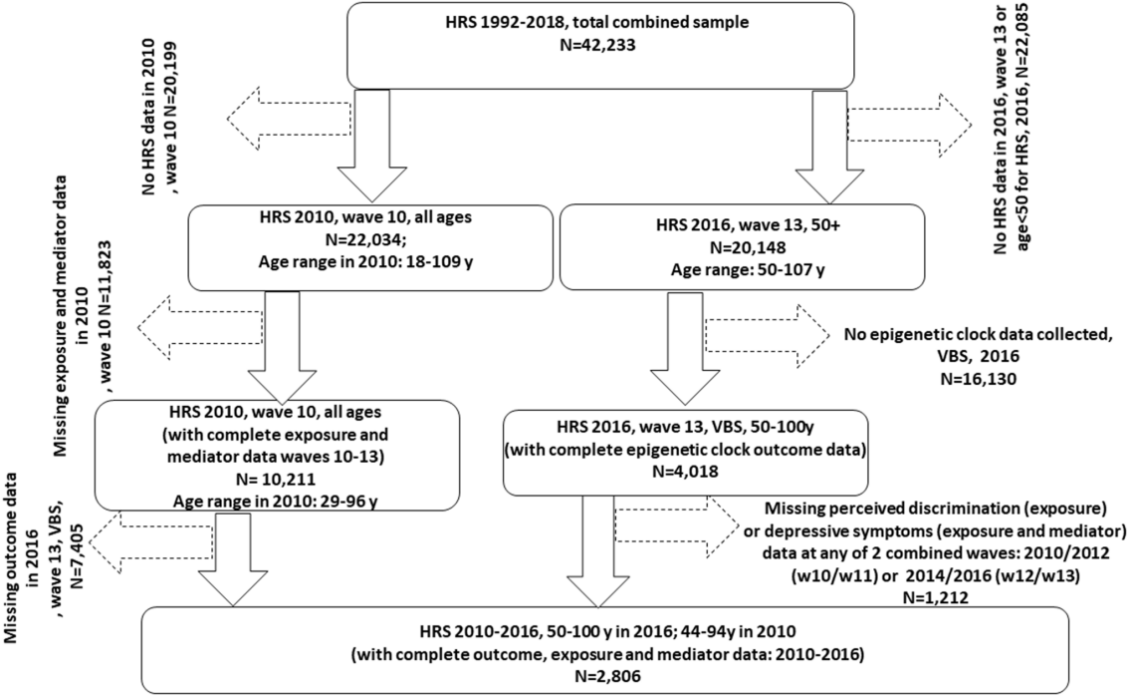
**Participant flowchart.** Abbreviations: HRS: Health and Retirement Study; N: Sample size; VBS: Venous Blood Study; w10: wave 10 (2010); w11: wave 11 (2012); w13: wave 13 (2014); w14: wave 14 (2016).

Our sample was restricted to HRS participants for whom data were collected during 2008, 2012 and 2016. The latest wave (2016) collected data on 4,108 participants by estimating 13 epigenetic clocks from DNAm and other biomarker data (Figure 1). We linked the latest release of 2016 epigenetic clock data with 2008 through 2016 EFTF respondent data using the 1992–2018 HRS longitudinal file developed by the RAND Center for the Study of Aging. Imputed data was used when possible, including household income data. Of 4,018 participants with complete QCed epigenetic clock data, 2,806 had complete 2010–2012 and 2014–2016 combined exposure and mediator (perceived discrimination scores and CES-D total score) data. This was the final sample size since all other covariates were imputed (<5% missing individually).

### DNA methylation data and epigenetic clocks

DNA methylation assays were done on a non-random subsample (*n* = 4,104) of HRS participants who consented to and participated in the 2016 VBS [3]. This subsample fully represents the entire HRS sample. Of those, 4,018 HRS participants had samples that passed quality control (QC).

DNAm data were based on assays done using the Infinium Methylation EPIC BeadChip at the University of Minnesota. Samples were randomized across plates by key demographic variables (i.e., age, cohort, sex, education, race/ethnicity) with 40 pairs of blinded duplicates. Analysis of duplicate samples showed a correlation >0.97 for all CpG sites. High quality methylation data is available for 97.9% of samples (*n* = 4,018). Prior to estimation of 13 clocks, missing beta methylation values were imputed with mean beta methylation values of probes across all samples. Details on data preprocessing and QC and a full description of the 13 epigenetic clocks are provided in Supplementary Method 1. Briefly, these 13 clocks were: (1) Horvath 1 [23]; (2) Hannum [24]; (3) Levine or PhenoAge [25]; (4) Horvath 2 [10]; (5) Lin [10]; (6) Weidner [26]; (7) VidalBralo [27]; (8) GrimAge [28]; (9) Yang [29]; (10) Zhang [30]; (11) Bocklandt [31]; (12) Garagnani [32]; (13) DunedinPoAm38 (MPOA) [28].

### Depressive symptoms

Depression symptomology was assessed using modified 8-item Center for Epidemiological Studies Depression Scale (CES-D), with higher scores reflecting higher levels of depression [33, 34] (Supplementary Method 2). In our present study, CES-D total score was used for combined 2010–2012 and 2014–2016 years to determine trajectories over time, and for combined years 2014–2016 as a potential mediator. It is worth noting that since CES-D is part of the core interview, the score was measured at the later year of the combined waves when available (i.e., 2012 or 2016). When, missing at those waves, it was measured in the earlier wave (i.e., 2010 or 2014).

### Experience of discrimination and reasons for perceived discrimination

#### Experience of discrimination, EOD

HRS respondents completed the abbreviated version of perceived everyday discrimination scale, which consists of 5 items assessing frequency of experiencing perceived everyday discrimination on a scale ranging from 1 (never) to 6 (almost every day). Items include the following: (a) “You are treated with less courtesy or respect than other people,” (b) “You receive poorer service than other people at restaurants or stores,” (c) “People act as if they think you are not smart,” (d) “People act as if they are afraid of you,” and (e) “You are threatened or harassed” and (f) “You receive poorer service or treatment than other people from doctors or hospitals” (Supplementary Method 2). This version of perceived everyday discrimination scale has demonstrated good reliability and validity and is used in studies on health among older Black adults [35, 36]. As a similar approach to previous work [35], we reverse-coded response items and summed over items to produce a continuous perceived everyday discrimination scale ranging from 6 to 36 (Cronbach’s alpha = 0.80). Higher scores indicate more frequent perceived everyday discrimination. This sum was re-scaled to zero by subtracting the final score by 6 (range: 0–30) in part of the analysis (*Med4way*). This score is hereafter named Experience of Discrimination or EOD and is described in other studies [19, 37, 38].

#### Reasons for perceived everyday discrimination, RPD

The HRS allows respondents to attribute perceived everyday discrimination to up to 11 reasons including age, ancestry, appearance, physical disability, race, sex, sexual orientation, weight, and other factors (Supplementary Method 2). See Supplementary Materials for more detailed breakdown of discrimination sources. We created a count for number of attributions HRS respondents offered for perceived everyday discrimination (range: 0–11). This score is hereafter named Reasons for Perceived Discrimination or RPD and is described in at least one other study [39]. In our present study, EOD and RPD scores were used for years 2010–2012 and 2014–2016 to determine trajectories over time, and for the combined years (2010–2012) as exposure in mediation models. Given that they were measured in half samples, around half of the final sample had data in 2010 and the other half in 2012. The same was the case for the 2014–2016 combined wave.

### Covariates

#### Socio-demographic characteristics

We accounted for sex (male, female), birth cohort, age, race/ethnicity (non-Hispanic White, non-Hispanic Black, Hispanic, Other) marital status (never married, married/partnered, separated/divorced, widowed), education (no degree, GED, high school graduate, some college, college degree or higher), work status (working, not working), federal insurance coverage (‘Yes’, ‘No’), total wealth (in U.S. dollars) (<25,000, 25,000–124,999, 125,000–299,999, ≥300,000) and number of household members (≤3, >3) [40]. Combined ages at 2010–2012 and 2014–2016 were used to determine trajectory exposure groups (see statistical analysis for details).

#### Lifestyle characteristics

We included smoking status (never smoker, past smoker, current smoker), frequency of alcohol consumption (abstinent, 1–3 days per month, 1–2 days per week, ≥3 days per week), and frequency of moderate/vigorous exercise (never, 1–4 times per month, >1 times per week).

#### Health characteristics

We classified self-rated health as “excellent/very good/good” and “fair/poor”. We also included self-reported measures of weight, height, and presence of cardiometabolic risk factors and chronic conditions as indicated by a physician. Aside from fixed covariates (e.g., sex and race), all other socio-demographic, lifestyle and health characteristics were included in our analyses as confounders measured at baseline year 2010. Moreover, these covariates underwent multiple imputations to maximize sample size after exclusion of missing data on exposures, mediators and outcomes between 2010 and 2016 (See statistical analysis for details).

### Statistical analysis

Using Stata 17.0 (StataCorp, College Station, TX, USA) [41], we accounted for sampling design complexity [42] by incorporating appropriate sampling weights, primary sampling units (*secu*) and strata (*stratum*). The sampling weight used was, as recommended, for the most limiting variables in the analysis. Therefore, we used the VBS sampling weight (*vbsi16wgtra*). Aside from epigenetic clock outcomes (the main determinant for the largest sample size), and the exposure and mediator variables (i.e., perceived discrimination and CES-D scores at the two combined visits of 2010–2012 and 2014–2016), baseline covariates measured in 2010 were multivariate-imputed with chained equations [43]. Consequently, population estimates of means, proportions and regression coefficients were obtained with Stata survey (svy) commands, computing standard errors (SE) with Taylor series linearization [42]. Comparison across sex and by racial/ethnic groups were made using svy:reg and svy:mlogit commands.

A Stata plugin (*traj* and *trajplot*) adapted from a well-established SAS procedure [44] was used to perform group-based trajectory modeling, GBTM – a specialized form of finite mixture modeling – to identify groups of older adults with similar developmental trajectories over time. This group-based approach utilizes a multinomial modeling strategy and maximum likelihood to estimate model parameters, with maximization achieved by the quasi-Newton procedure. We specified a zero-inflated Poisson (*zip*) for the selected outcomes, with intercept (0), linear (1), quadratic (2) and cubic (3) orders for each group trajectory and displayed group-based trajectories over time with 95% confidence intervals (CI). For consistency and ease of interpretation, we defined up to three groups per outcome. We reported the Akaike Information Criterion (AIC) for each group-based trajectory model as a goodness-of-fit measure. This procedure was applied to three main scores, hereafter labelled as trajectory exposures: (1) Perceived discrimination score trajectory between 2010–2012 and 2014–2016; (2) Reasons for Perceived discrimination score trajectory for the same two periods; (3) CES-D total score for the same two periods. Age was used as the time variable in these models.

To test our main hypotheses, we ran a series of ordinary least square linear regression models, looping over 13 epigenetic clock outcomes and the 3 trajectory exposures, entered as categorical variables (binary or 3-level, taking the lowest risk category as the referent), and adjusting for potentially confounding covariates in sequential manner. Model 1 adjusted for age at follow-up (2016), birth cohort, sex and race/ethnicity; Model 2 further adjusted Model 1 for income and education; Model 3 further adjusted Model 2 for all the remaining lifestyle and health-related factors. These associations were tested first in the overall sample. They were also tested in stratified analyses by sex and race (Non-white, White), separately, if two-way interaction terms between sex/race and each trajectory exposure were indicative of heterogeneity in effects.

Continuous CES-D score measured in 2014–2016 combined wave was also tested as a potential mediator/moderator in the association of perceived discrimination scores (2010–2012) with biological aging (2016) as measured by 13 epigenetic clocks. Specifically, the overall effect of each main perceived discrimination exposure on biological aging, in the presence of a mediator with which the exposure may interact, was decomposed into four distinctive components: (i) neither mediation nor interaction; (ii) interaction alone (and not mediation); (iii) both mediation and interaction; and (iv) only mediation (but not interaction). This four-way decomposition unifies methods to attribute effects to interactions and methods that assess mediation. It has recently been introduced in Stata, allowing to estimate four-way decomposition using parametric or semi-parametric regression models. Importantly, *Med4way* command [45] (https://github.com/anddis/med4way) was used to test mediation and interaction of the total effect of perceived discrimination exposures on the 13 epigenetic clocks with CES-D total score as the potential mediator/moderator, using OLS linear models for the outcome and each mediator/moderator. Four-way decomposition was applied to the total sample, and among men and women, separately, as well as by race (White vs. Non-White), combining findings from 5 imputations using Rubin’s rule [46]. Both the full (adjusted for all covariates as exogenous variables) and reduced (adjusted only for basic demographics) models were presented, focusing on findings from the full model. Type I error was set at 0.05 for all analyses and corrected for multiplicity of exposure/mediator types (total of 3) for minimally adjusted models (i.e., Model 1), using familywise Bonferroni correction, with the corrected *p*-value being set to 0.05/3 = 0.017.

As a sensitivity analysis, a structural equations model was performed whereby the outcomes were alternatively one of each 13 epigenetic clocks, the two main exposures were EOD an RPD measured in 2010-2012, and the potential mediator was CES-D total score measured in 2014–2016. Exogenous variables included in this model were allowed to predict each of the outcome, exposure and mediator, and those were 2014–2016 age, sex, and race (Non-White vs. White). Total effects were examined for statistical significance at type I error of 0.05 and were decomposed into indirect and direct effect. Statistically significant mediation was determined when an indirect effect going in the same direction as a statistically significant total effect, had an associated *p*-value < 0.05. More details regarding this approach is provided in an earlier study [47]. This analysis was conducted on the first imputation of five and was compared to at least one other imputation.

## RESULTS

Table 1 presents baseline socio-demographic, lifestyle and health-related characteristics using data from 2,806 HRS participants (55.6% female; mean (±SEM) age in 2016: 69.3 ± 0.3 years; 82.3% NHW), with proportion of female significantly greater among non-NHW adults vs. NHW adults (62.7% vs. 54.1%). Sex and race/ethnic differences were observed in key characteristics, including age, whereby non-NHW were on average younger than NHW adults by ~2 years in 2016 (*p* = 0.006), while non-NHW and females tended to have a lower educational attainment and income compared to NHW and male adults, respectively, and were less likely to be married/partnered, to be physically active, to consume alcohol ≥3 days a week, and were more likely than their counterparts to be living in a larger household, and have a greater mean number of co-morbidities. While a greater proportion of males were working, they also had a higher proportion of current smokers, and more prevalent heart disease in 2010, compared to female participants. Both BMI and self-rated health differed by race/ethnicity and not by sex, whereby poorer health and greater mean BMI was observed among non-NHW vs. NHW adults.

**Table 1:** Study sample socio-demographic, lifestyle and health-related characteristics by sex and by race, HRS 2010-2016^a^.

	**Overall**	**Males**	**Females**	**NHW**	**Non-NHW**	**P^b^_sex_**	**P^b^_race_**
	**Mean/% ± SE**	**Mean/% ± SE**	**Mean/% ± SE**	**Mean/% ± SE**	**Mean/% ± SE**		
SOCIO-DEMOGRAPHIC							
***Sex:***							
Male	44.4 ± 1.5	100.0	0.0	45.9 ± 1.7	37.3 ± 2.7	__	0.016
Female	55.6 ± 1.5	0.0	100.0	54.1 ± 1.7	62.7 ± 2.7	__	Ref
***Age (years), 2010***							
Mean ± SEM	63.4 ± 0.3	63.0 ± 0.4	63.7 ± 0.3	63.7 ± 0.36	61.9 ± 0.5	0.16	0.004^d^
***Age (years), 2012***							
Mean ± SEM	65.2 ± 0.3	64.8 ± 0.4	65.5 ± 0.3	65.5 ± 0.3	63.6 ± 0.5	0.15	0.003^d^
***Age (years), 2016***							
Mean ± SEM	69.2 ± 0.3	68.9 ± 0.4	69.5 ± 0.3	69.6 ± 0.36	67.6 ± 0.5	0.15	0.003^d^
***Birth cohort:***							
Original/AHEAD/Children of the Depression	27.4 ± 1.3	25.7 ± 1.8	28.8 ± 1.6	29.2 ± 1.5	18.7 ± 2.2	Ref	Ref
War Babies	20.6 ± 1.0	21.4 ± 1.8	20.0 ± 1.3	20.1 ± 1.1	23.0 ± 2.2	0.26	<0.001
Early Baby Boomers	25.2 ± 1.0	26.8 ± 1.9	23.9 ± 1.3	24.3 ± 1.2	29.5 ± 2.4	0.11	0.003
Mid Baby Boomers	26.8 ± 1.4	26.1 ± 2.2	27.3 ± 1.4	26.3 ± 1.5	28.9 ± 2.2	0.61	0.007
***Race:***							
Non-Hispanic White	82.3 ± 1.4	85.7 ± 1.8	80.8 ± 1.7	100.0	0.0	Ref	__
Non-Hispanic black, African descent	8.5 ± 0.7	7.1 ± 1.0	9.5 ± 0.8	__	49.7 ± 3.5	0.035	__
Hispanic	6.1 ± 1.0	5.6 ± 1.1	6.6 ± 1.2	__	36.0 ± 3.8	0.23	__
Other	2.4 ± 0.4	1.6 ± 0.4	3.1 ± 0.6	__	14.3 ± 1.9	0.056	__
***Education:***							
No degree	9.2 ± 0.6	8.4 ± 0.9	10.0 ± 0.9	5.9 ± 0.5	25.3 ± 2.3	0.010^d^	<0.001^d^
GED	4.8 ± 0.5	5.2 ± 0.8	4.5 ± 0.7	4.4 ± 0.5	6.7 ± 1.3	0.60	0.002^d^
High School graduate	28.2 ± 1.0	24.7 ± 1.3	31.0 ± 1.5	28.2 ± 1.1	28.1 ± 2.1	0.001^d^	0.001^d^
Some college	27.8 ± 1.0	26.6 ± 1.5	28.7 ± 1.5	28.8 ± 1.3	22.5 ± 1.7	0.010^d^	0.040^d^
College degree or higher	30.1 ± 1.4	35.1 ± 2.1	26.1 ± 1.7	32.7 ± 1.6	17.5 ± 2.1	Ref	Ref
***Marital status:***							
Never married	6.7 ± 0.7	7.1 ± 1.1	6.4 ± 0.8	5.9 ± 0.7	10.9 ± 1.6	0.49	<0.001^d^
Married/Partnered	70.3 ± 1.2	80.2 ± 1.6	62.4 ± 1.6	72.9 ± 1.3	57.2 ± 2.9	Ref	Ref
Separated/Divorced	12.9 ± 0.9	9.5 ± 1.1	15.5 ± 1.2	11.5 ± 0.9	19.3 ± 2.0	<0.001^d^	<0.001^d^
Widowed	10.2 ± 0.6	3.3 ± 0.6	15.6 ± 0.9	9.6 ± 0.7	12.6 ± 1.7	<0.001^d^	0.005^d^
***Work status:***							
Not Working	49.4 ± 1.4	45.4 ± 1.9	52.6 ± 1.7	48.4 ± 1.6	54.1 ± 2.8	0.004^d^	0.091^d^
Working	50.6 ± 1.4	54.6 ± 1.9	47.4 ± 1.7	51.6 ± 1.6	46.0 ± 2.8	Ref	Ref
***Federal health insurance coverage:***							
No	53.0 ± 1.5	53.4 ± 2.1	52.7 ± 1.5	53.4 ± 1.7	51.2 ± 2.8	Ref	Ref
Yes	47.0 ± 1.5	46.6 ± 2.1	47.3 ± 1.5	46.6 ± 1.7	48.8 ± 2.8	0.74	0.51^d^
***Total wealth ($):***							
< 25,000	20.3 ± 1.2	14.0 ± 1.3	25.4 ± 1.5	15.7 ± 1.1	43.0 ± 2.3	<0.001^d^	<0.001^d^
25,000–124,999	62.5 ± 1.1	66.5 ± 1.8	59.3 ± 1.4	65.3 ± 1.2	48.7 ± 2.4	Ref	Ref
125,000–299,999	15.0 ± 0.8	17.1 ± 1.4	13.2 ± 1.1	16.5 ± 1.0	7.5 ± 1.3	0.32	0.035
≥ 300,000	2.1 ± 0.4	2.1 ± 0.6	2.0 ± 0.5	2.3 ± 0.4	0.0	0.87	0.097
***Number of household members:***							
≤3	88.0 ± 0.9	85.6 ± 1.4	89.9 ± 1.1	90.3 ± 0.8	76.9 ± 3.0	Ref	Ref
>3	12.0 ± 0.9	14.4 ± 1.4	10.1 ± 1.1	9.7 ± 0.8	23.1 ± 3.0	0.017^d^	<0.001^d^
**LIFESTYLE:**							
***Smoking status:***							
Never smoker	44.9 ± 1.2	37.6 ± 1.7	50.7 ± 1.7	44.4 ± 1.4	47.2 ± 2.4	Ref	Ref
Past smoker	43.2 ± 1.1	49.9 ± 1.7	37.9 ± 1.6	44.2 ± 1.3	38.2 ± 3.6	<0.001^d^	0.13
Current smoker	11.9 ± 0.7	12.4 ± 1.2	11.5 ± 0.8	11.3 ± 0.7	14.5 ± 1.7	<0.001^d^	0.27
***Frequency of alcohol consumption:***							
Abstinent	36.1 ± 1.1	30.7 ± 1.8	40.5 ± 1.3	34.4 ± 1.3	44.8 ± 2.7	Ref	Ref
1–3 days per month	21.6 ± 1.0	18.8 ± 1.3	23.8 ± 1.3	20.8 ± 1.1	25.4 ± 2.0	0.71	0.62
1–2 days per week	25.6 ± 0.9	29.3 ± 1.7	22.6 ± 1.1	26.1 ± 1.1	22.9 ± 2.1	<0.001^d^	0.022^d^
≥3 days per week	16.7 ± 1.0	21.2 ± 1.9	13.0 ± 1.1	18.7 ± 1.1	6.8 ± 1.2	<0.001^d^	<0.001^d^
***Frequency of moderate/vigorous physical exercise:***							
Never	13.0 ± 0.7	8.7 ± 1.1	16.5 ± 1.1	12.2 ± 0.8	17.1 ± 1.6	<0.001^d^	0.001^d^
1–4 times per month	26.1 ± 1.0	27.4 ± 1.4	25.1 ± 1.4	24.9 ± 1.1	32.0 ± 2.3	0.98	0.001^d^
>1 times per week	60.9 ± 1.1	63.9 ± 1.7	58.4 ± 1.6	62.9 ± 1.1	51.0 ± 2.5	Ref	Ref
**HEALTH**-**RELATED:**							
***Self-rated health:***							
Excellent/very good/good	82.5 ± 0.9	83.7 ± 1.2	81.5 ± 1.1	85.4 ± 0.9	68.4 ± 2.4	Ref	Ref
Fair/poor	17.5 ± 0.9	16.3 ± 1.2	18.5 ± 1.2	14.6 ± 0.9	31.6 ± 2.4	0.18	<0.001^d^
***Body mass index (kg/m^2^):***							
Mean ± SEM	28.9 ± 0.2	29.2 ± 0.2	28.7 ± 0.2	28.7 ± 0.2	29.8 ± 0.3	0.086	0.003^d^
***Cardiometabolic risk factors and chronic conditions:***							
*Hypertension:*							
No	49.8 ± 1.4	49.5 ± 1.8	50.0 ± 1.6	51.8 ± 1.6	40.0 ± 2.7	0.80	0.001^d^
Yes	50.2 ± 1.4	50.5 ± 1.8	50.0 ± 1.6	48.2 ± 1.6	60.0 ± 2.7	Ref	Ref
*Diabetes:*
No	83.1 ± 0.9	81.6 ± 1.2	84.3 ± 1.2	84.8 ± 0.9	74.9 ± 1.9	Ref	Ref
Yes	16.9 ± 0.9	18.4 ± 1.2	15.7 ± 1.2	15.2 ± 0.9	25.1 ± 2.0	0.079^d^	<0.001^d^
*Heart disease:*							
No	83.0 ± 1.0	80.3 ± 1.6	85.1 ± 1.1	82.6 ± 1.1	84.8 ± 1.6	Ref	Ref
Yes	17.0 ± 1.0	19.7 ± 1.6	14.9 ± 1.1	17.4 ± 1.1	15.3 ± 1.6	0.008^d^	0.28
*Stroke:*							
No	95.2 ± 0.4	94.9 ± 0.5	95.3 ± 0.6	95.4 ± 0.4	94.1 ± 1.2	0.63	Ref
Yes	4.8 ± 0.4	5.1 ± 0.5	4.7 ± 0.6	4.6 ± 0.4	5.9 ± 1.2	Ref	0.28
***Number of conditions***							
Mean ± SEM	0.89 ± 0.02	0.94 ± 0.03	0.85 ± 0.03	0.85 ± 0.03	1.06 ± 0.04	0.014^d^	<0.001^d^

Abbreviations: SE: Standard Error; NHW: Non-Hispanic White; Ref: Referent category; SEM: Standard Error of the Mean. ^a^Values are means ± SE or % ± SE, overall and across sex or race/ethnicity groups for main baseline and fixed sample characteristics (See Covariates section for detail), taking into account sampling weights and sampling design complexity in multiple imputed data. All covariates are measured in 2010 unless stated otherwise. ^b^Based on linear or multinomial logit models with sex or race as the only predictors of continuous and categorical variables, respectively, taking into account sampling weights and sampling design complexity in multiple imputed data. Italicized findings have *p* < 0.10 but >0.05. ^c^Number of chronic conditions among hypertension, diabetes, heart disease and stroke. ^d^*P* < 0.05 after further adjustment of other demographic variables, including age in 2016, birth cohort, sex and race.

**Figure 2:**
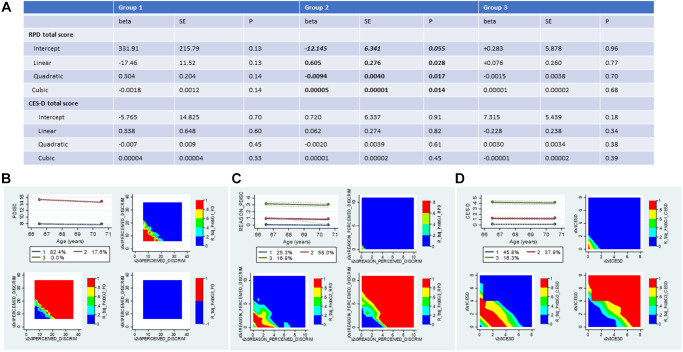
**Trajectories in perceived discrimination and depressive symptoms measures 2010-2016: Group-based trajectory models; HRS 2010-2016 (*N* = 2,806).** (**A**) Results of the Group-based trajectory model for RPD and CES-D score; (**B**) Trajectory plot for EOD and contour plots for raw values at each combined visit per group; (**C**) Trajectory plot for RPD and contour plots for raw values at each combined visit per group; (**D**) Trajectory plot for CES-D and contour plots for raw values at each combined visit per group. Abbreviations: EOD: Experience of Discrimination; HRS: Health and Retirement Study; RPD: Reasons for perceived discrimination; PDISC: same as EOD and PERCEIVED_DISCRIM; REASON_PDISC: same as RPD and REASON_PERCEIVED_DISCRIM; v0: baseline visit, wave 10 (2010); v1: first follow-up visit, wave 11 (2012); v2: second follow-up visit, wave 12 (2014); v3: third follow-up visit, wave 13 (2016); v0v1: combined visits 0 and 1; v2v3: combined visits 2 and 3. Note: v0v1AGE was mainly v1AGE unless v1AGE was missing, then it was imputed with v0AGE. Similarly, v2v3AGE was mainly v3AGE unless v3AGE was missing, then it was imputed with v2AGE. The same applied to the CES-D scores, whereby v0v1CESD was mainly v1CESD, and v2v3CESD was mainly v3CESD. EOD for combined v0v1 was half in v0 and the other half in v1, and similarly for RPD, given that they were measured in half samples. The Table is based on zero-inflated Poisson GBTM models. Predicted values for each score at each age, require exponentiation of the linear combinations.

Group-based trajectory model (GBTM) results indicated that only 2 of 3 exposures could be grouped using a model for 3-group membership that included a linear, quadratic, and cubic term for age, namely RPD and CES-D total score using zero-inflated Poisson regression models. Figure 2 shows the mean posterior probabilities assigned to each group for each of these three variables (2B, 2C and 2D). EOD was assigned 2 groups using a non-parametric process. RPD and CES-D total score trajectories were assigned 3 groups using the models shown in 2A. In addition to posterior probabilities, actual group membership was estimated and used in subsequent analyses (Supplementary Tables 1 and 2).

Based on Supplementary Table 1, point and trajectory exposures were patterned by sex and race/ethnicity, with greater likelihood of depressive symptoms (DEP) and elevated scores on extent of and reasons for perceived discrimination (EOD and RPD) observed among females and non-NHW across waves. Similarly, epigenetic age was on average greater among males for 7 of 13 EPICLOCK measures, while many of these measures indicated that epigenetic age was lower among non-NHW adults vs. NHW adults, including the Horvath, Horvath 2, Levine (PhenoAge) and Hannum EPICLOCK measures. Upon adjustment for chronological age, additional significant contrasts were detected indicating that accelerated epigenetic aging occurred at different rates across sex and race/ethnicity groups. The 13 EPICLOCK measures are presented in a matrix of scatter plots in Supplementary Figure 1 along with chronological age measured in 2016 (end of wave). In general, all clocks were shown to be positively associated with chronological age, with the exception of the Bocklandt EPICLOCK, which as expected, is inversely related to age [3, 31].

Table 2 and Supplementary Table 2 report results from a series of multiple OLS linear regression models examining the associations of the three trajectory exposures (EOD, RPD and CES-D total scores) with 13 markers of epigenetic aging. Upon correction for multiple testing in reduced model 1 (adjusted for age in 2016, birth cohort, sex, and race/ethnicity), among females, a sustained high EOD score was associated with epigenetic age acceleration based on the VIDAL-BRALO DNAmage marker (Supplementary Table 2, Model 1: β = +1.059 ± 0.420, *p* = 0.015). This association was somewhat attenuated upon adjustment for socio-economic status variables (Supplementary Table 2, Model 2: β = +0.914 ± 0.420, *p* = 0.034), but markedly attenuated with further adjustment for lifestyle and health-related factors including smoking and BMI (Supplementary Table 2, Model 2: β = +0.697 ± 0.443, *p* = 0.12). RPD trajectory was not associated with epigenetic age measures overall (Table 2) or within groups (Supplementary Table 2), upon correction for multiple testing.

**Table 2:** Trajectories in experience of discrimination, reasons for perceived discrimination and depressive symptoms (2010-2016) and their association with 13 epigenetic clocks (2016): Multiple OLS linear regression models, overall: HRS 2010-2016^a,b^.

**Y = Epigenetic clock**	**X = Experience of discrimination (EOD) score trajectory**		**X = Reasons for perceived discrimination (RPD) trajectory**				**X = CES-D total score trajectory**			
	**High vs. Low**		**Medium vs. Low**		**High vs. Low**		**Medium vs. Low**		**High vs. Low**	
	**β ± SE**	***P***	**β ± SE**	***P***	**β ± SE**	***P***	**β ± SE**	***P***	**β ± SE**	***P***
HORVATH DNAmage										
Model 1	−0.129 ± 0.378	0.75	+0.332 ± 0.332	0.32	−0.202 ± 0.395	0.61	−0.033 ± 0.311	0.92	−0.605 ± 0.420	0.16
Model 2	−0.162 ± 0.369	0.66	+0.304 ± 0.335	0.37	−0.236 ± 0.388	0.55	−0.059 ± 0.295	0.84	−0.634 ± 0.391	0.11
Model 3	−0.400 ± 0.403	0.33	+0.299 ± 0.329	0.37	−0.557 ± 0.393	0.16	−0.157 ± 0.311	0.62	−0.971 ± 0.423	0.026
HANNUM DNAmage										
Model 1	−0.077 ± 0.275	0.78	−0.059 ± 0.259	0.82	−0.331 ± 0.338	0.33	+0.115 ± 0.244	0.64	+0.759 ± 0.354	0.037
Model 2	−0.170 ± 0.261	0.52	−0.099 ± 0.262	0.71	−0.434 ± 0.341	0.21	+0.047 ± 0.238	0.84	*+0.597 ± 0.344*	*0.088*
Model 3	−0.332 ± 0.275	0.23	−0.172 ± 0.250	0.49	−0.786 ± 0.356	0.032	−0.062 ± 0.247	0.80	+0.306 ± 0.349	0.38
LEVINE DNAmage										
Model 1	−0.238 ± 0.488	0.63	−0.109 ± 0.340	0.75	−0.301 ± 0.467	0.52	+1.182 ± 0.434	0.009^c^	+1.114 ± 0.543	0.045
Model 2	0.430 ± 0.505	0.40	−0.167 ± 0.331	0.62	−0.483 ± 0.473	0.31	+1.064 ± 0.428	0.016	+0.794 ± 0.545	0.15
Model 3	−0.623 ± 0.490	0.21	−0.288 ± 0.318	0.37	−0.933 ± 0.446	0.041	+0.926 ± 0.427	0.035	+0.430 ± 0.636	0.50
HORVATH 2 DNAmage										
Model 1	+0.390 ± 0.270	0.16	+0.316 ± 0.234	0.18	+0.284 ± 0.373	0.45	−0.189 ± 0.246	0.45	+0.358 ± 0.320	0.27
Model 2	+0.289 ± 0.263	0.28	+0.262 ± 0.234	0.27	+0.174 ± 0.377	0.65	−0.255 ± 0.241	0.30	+0.206 ± 0.316	0.52
Model 3	+0.123 ± 0.275	0.66	+0.228 ± 0.230	0.33	−0.060 ± 0.382	0.88	−0.345 ± 0.240	0.16	−0.067 ± 0.306	0.83
LIN DNAmage										
Model 1	+0.193 ± 0.472	0.68	+0.077 ± 0.437	0.87	−0.104 ± 0.547	0.86	+1.226 ± 0.325	<0.001^c^	+0.605 ± 0.452	0.19
Model 2	+0.238 ± 0.473	0.63	+0.040 ± 0.435	0.93	−0.125 ± 0.562	0.83	+1.309 ± 0.318	<0.001	*+0.841 ± 0.468*	*0.078*
Model 3	+0.067 ± 0.465	0.89	−0.040 ± 0.428	0.93	−0.550 ± 0.512	0.29	+1.233 ± 0.321	<0.001	+0.496 ± 0.499	0.33
WEIDNER DNAmage										
Model 1	−0.135 ± 0.621	0.83	−0.718 ± 0.576	0.22	−0.912 ± 0.818	0.27	+0.632 ± 0.547	0.25	+0.922 ± 0.795	0.25
Model 2	−0.065 ± 0.631	0.92	−0.678 ± 0.580	0.25	−0.917 ± 0.795	0.25	+0.712 ± 0.556	0.21	+0.970 ± 0.556	0.21
Model 3	+0.077 ± 0.642	0.91	−0.796 ± 0.567	0.17	−1.110 ± 0.840	0.19	+0.877 ± 0.582	0.14	+1.147 ± 0.884	0.20
VIDAL−BRALO DNAmage										
Model 1	+0.093 ± 0.358	0.80	−0.019 ± 0.269	0.94	+0.092 ± 0.452	0.84	+0.294 ± 0.227	0.20	+0.439 ± 0.296	0.14
Model 2	+0.049 ± 0.359	0.89	−0.029 ± 0.264	0.91	+0.027 ± 0.441	0.95	+0.282 ± 0.225	0.22	+0.359 ± 0.309	0.25
Model 3	−0.093 ± 0.341	0.79	−0.133 ± 0.257	0.61	−0.306 ± 0.458	0.51	+0.181 ± 0.221	0.42	+0.011 ± 0.347	0.97
YANG DNAmage										
Model 1	−0.000 ± 0.001	0.95	−0.0003 ± 0.0009	0.76	−0.0017 ± 0.0009	0.76	*+0.001 ± 0.001*	*0.090*	+0.003 ± 0.001	0.007^c^
Model 2	−0.000 ± 0.001	0.60	−0.0003 ± 0.0009	0.73	−0.0020 ± 0.0010	0.047	+0.0010 ± 0.0008	0.19	*+0.0019 ± 0.0010*	*0.078*
Model 3	−0.0008 ± 0.0008	0.37	−0.0004 ± 0.0009	0.65	−0.0024 ± 0.0011	0.030	+0.0007 ± 0.0008	0.38	+0.0010 ± 0.0011	0.36
ZHANG DNAmage										
Model 1	+0.019 ± 0.031	0.55	−0.015 ± 0.022	0.51	+0.004 ± 0.0295	0.91	+0.089 ± 0.025	0.001^c^	+0.128 ± 0.026	<0.001^c^
Model 2	−0.003 ± 0.031	0.91	−0.021 ± 0.022	0.34	−0.014 ± 0.030	0.63	+0.073 ± 0.024	0.005	+0.087 ± 0.026	0.001
Model 3	−0.025 ± 0.027	0.36	−0.030 ± 0.020	0.15	−0.042 ± 0.029	0.15	+0.048 ± 0.024	0.049	+0.041 ± 0.026	0.13
BOCKLANDT DNAmage										
Model 1	+0.005 ± 0.004	0.19	−0.0021 ± 0.0026	0.42	+0.0007 ± 0.0044	0.88	−0.0036 ± 0.0036	0.32	−0.0011 ± 0.0052	0.83
Model 2	−0.006 ± 0.004	0.14	−0.0018 ± 0.0025	0.49	+0.0014 ± 0.0043	0.74	−0.0038 ± 0.0037	0.31	−0.0012 ± 0.0053	0.83
Model 3	*+0.008 ± 0.003*	*0.054*	−0.0010 ± 0.0025	0.69	+0.0028 ± 0.0044	0.53	−0.0022 ± 0.0039	0.57	+0.0018 ± 0.0056	0.76
GARAGNANI DNAmage										
Model 1	+0.002 ± 0.003	0.43	−0.002 ± 0.004	0.56	+0.006 ± 0.004	0.20	+0.0025 ± 0.002	0.30	+0.0071 ± 0.0046	0.13
Model 2	+0.001 ± 0.003	0.68	−0.002 ± 0.004	0.53	+0.005 ± 0.004	0.26	+0.0019 ± 0.003	0.45	+0.0050 ± 0.0045	0.28
Model 3	+0.0001 ± 0.003	0.98	−0.0029 ± 0.0035	0.42	+0.0041 ± 0.0042	0.34	+0.0016 ± 0.0025	0.53	+0.0037 ± 0.0047	0.44
DNAm GRIMAGE										
Model 1	+0.310 ± 0.282	0.28	−0.075 ± 0.251	0.77	+0.243 ± 0.333	0.47	+0.742 ± 0.236	0.003^c^	1.831 ± 0.327	<0.001^c^
Model 2	−0.046 ± 0.274	0.87	−0.189 ± 0.220	0.40	−0.067 ± 0.332	0.84	+0.502 ± 0.220	0.026	1.220 ± 0.322	<0.001
Model 3	−*0.404 ± 0.225*	*0.079*	−0.272 ± 0.186	0.15	−0.205 ± 0.273	0.46	+0.032 ± 0.190	0.87	*+0.517 ± 0.300*	*0.089*
MPOA										
Model 1	+0.003 ± 0.006	0.60	+0.001 ± 0.005	0.81	+0.005 ± 0.006	0.38	+0.008 ± 0.005	0.13	+0.020 ± 0.007	0.004^c^
Model 2	−0.001 ± 0.006	0.83	−0.0001 ± 0.0045	0.97	+0.0013 ± 0.0065	0.84	+0.0043 ± 0.005	0.40	+0.011 ± 0.007	0.10
Model 3	−0.006 ± 0.006	0.29	−0.0015 ± 0.0040	0.72	−0.0008 ± 0.0056	0.89	−0.0021 ± 0.0048	0.66	+0.0016 ± 0.007	0.82

Abbreviations: CES-D: Centers for Epidemiological Studies-Depression; DNAm: DNA methylation; DNAmage: DNA methylation age; GBTM: Group-based trajectory models; HRS: Health and Retirement Study; NHW: Non-Hispanic White. See Supplementary Methods for epigenetic clock abbreviations. ^a^OLS regression models with epigenetic clocks as alternative outcomes and trajectories in EOD, RPD and CES-D scores as alternative exposures. Subpopulation sample size *N* = 2,728, accounting for sampling weights, PSU and strata. Aside from fixed covariates and age which is measured in 2016, all other covariates were measured in 2010. Stratified analysis by sex and/or race was presented only when *p* < 0.05 for Exposure^*^sex or Exposure^*^race for at least one contrast in the unstratified model with 2-way interaction terms. ^b^Model 1 adjusted for sex, age in 2016, birth cohort and race/ethnicity; Model 2 further adjusted Model 1 for education and total wealth in 2010; Model 3 further adjusted Model 2 for the remaining socio-demographic, lifestyle and health-related factors (See Covariates section for detail). Italicized findings have *p* < 0.10 but >0.05. ^c^Passed correction for multiple testing at type I error of 0.05 (corrected *p*-value accounting for exposure type: 0.017), applied only to Model 1.

More importantly, among females in Supplementary Table 2, having a moderate or high CES-D total score over time was associated with faster epigenetic age acceleration based on the LIN DNAmage marker in the both models 1 and 2. This association remained largely unaltered in Model 3, particularly in the moderate CES-D group, indicating a 1.8–1.9 y greater epigenetic age compared to the sustained lower CES-D group (*P* < 0.001). Moreover, overall (Table 2), both the YANG and ZHANG DNAmage measures were found to be higher in the “sustained moderate and/or high CES-D total score” groups vs. “sustained low”, with a dose-response relationship, upon adjustment for chronological age, birth cohort, sex and race/ethnicity (*P* < 0.017 for at least one contrast). Nevertheless, these relationships were markedly attenuated for the YANG DNAmage clock after addition of SES factors into the model. These relationships were attenuated for both measures upon further adjustment for lifestyle and health-related factors (Table 2, Model 3: *P* > 0.05 for high vs. low in both YANG and ZHANG DNAmage measures), indicating potential confounding and/or mediation by SES, lifestyle and health-related characteristics. Similar patterns were found for GrimAge and MPOA markers, whereby adjustment for lifestyle and health-related factors attenuated the positive association between sustained elevated CES-D and epigenetic aging as measured by these two markers, overall (Table 2) and among NHW for MPOA (Supplementary Table 2) and in both racial/ethnic groups for GrimAge (Table 2 and Supplementary Table 2).

Tables 3, 4 and Supplementary Tables 3, 4 show findings from reduced and full 4-way mediation analyses, with reduced model including only age, sex, birth cohort and race/ethnicity (Non-White vs. White) as potential exogenous variables. The full model added all other potentially confounding 2010 covariates as exogenous (See Covariate section). Overall and/or among female and/or NHW participants, higher RPD in 2010–2012 had significant adverse total effects on epigenetic age acceleration in 2016, based on GrimAge, MPOA, Levine (PhenoAge) and Horvath 2 clocks, with 20–50% of these effects being explained by a pure indirect effect though CES-D total score in 2014–2016 (Supplementary Table 4). Many of these total effects were attenuated upon addition of socio-economic, lifestyle and health-related factors (Table 4). One notable finding in the full model (Table 4), however, was that among females, RPD remained associated with epigenetic aging based on the GrimAge measure (TE = +0.171, *p* = 0.008), an effect that was largely a direct one (CDE = +189, *p* = 0.015), independent of the mediating effect of DEP. In contrast, no statistically significant total effect of EOD (2010–2012) on measures of epigenetic aging was detected overall or within sex or race/ethnicity strata (Table 3 and Supplementary Table 3). Despite an undetected total effect between EOD and the Levine (PhenoAge) clock, there was a pure indirect effect that was detected through DEP, particularly among NHW (Table 4 and Supplementary Table 3). A similar pattern was observed for PhenoAge and RPD (Table 4 and Supplementary Table 4).

**Table 3:** Experience of discrimination (EOD: 2010/2012) → depressive symptoms (CES-D: 2014/2016) → epigenetic clocks (2016): 4-way mediation analysis, overall and by sex and race, full model: HRS 2010-2016.

**Y = Epigenetic clock**	**Overall**		**Males**		**Females**		**NHW**		**Non-Whites**	
	**β ± SE**	***P***	**β ± SE**	***P***	**β ± SE**	***P***	**β ± SE**	***P***	**β ± SE**	***P***
HORVATH DNAmage										
TE	−0.0192 ± 0.0342	0.58	−0.187829 ± 0.137934	0.17	−0.023891 ± 0.046819	0.61	−0.019190 ± 0.041975	0.65	−0.012652 ± 0.059080	0.83
CDE	−0.0123 ± 0.0396	0.76	−0.200620 ± 0.163653	0.22	−0.018848 ± 0.055332	0.73	−0.010741 ± 0.047441	0.82	−0.008605 ± 0.072629	0.91
INTREF	−0.0043 ± 0.0123	0.73	0.047560 ± 0.062331	0.45	−0.005333 ± 0.018673	0.78	−0.0048 ± 0.013329	0.72	−0.004931 ± 0.028834	0.86
INTMED	−0.00035 ± 0.00101	0.73	0.00876 ± 0.01160	0.45	−0.000404 ± 0.001415	0.78	−0.000443 ± 0.001226	0.72	−0.000319 ± 0.001864	0.86
PIE	−0.00227 ± 0.0075	0.76	−0.043527 ± 0.028797	0.13	0.000694 ± 0.009667	0.94	−0.003186 ± 0.008820	0.72	0.00120 ± 0.014586	0.93
HANNUM DNAmage										
TE	−0.029072 ± 0.027427	0.29	−0.010186 ± 0.111398	0.93	−0.033330 ± 0.037244	0.37	−0.005189 ± 0.033813	0.88	−0.069365 ± 0.046238	0.13
CDE	−0.04063 ± 0.031710	0.20	0.01922 ± 0.132428	0.89	−0.049480 ± 0.044014	0.26	−0.012520 ± 0.038221	0.74	−0.093600 ± 0.056886	0.10
INTREF	0.006002 ± 0.009899	0.54	−0.040959 ± 0.050383	0.42	0.011753 ± 0.014865	0.43	0.002005 ± 0.010741	0.85	0.01723 ± 0.022632	0.45
INTMED	0.000493 ± 0.000814	0.55	−0.00754 ± 0.009379	0.42	0.000890 ± 0.001131	0.43	0.000184 ± 0.000987	0.85	0.001113 ± 0.001473	0.45
PIE	0.005065 ± 0.006033	0.40	0.019094 ± 0.022529	0.40	0.003507 ± 0.007707	0.65	0.005141 ± 0.007124	0.47	0.005892 ± 0.011458	0.61
LEVINE DNAmage										
TE	−0.006324 ± 0.036060	0.86	−0.070358 ± 0.143741	0.63	−0.01731 ± 0.04945	0.73	0.034101 ± 0.0442640	0.44	−0.092633 ± 0.061732	0.13
CDE	0.001611 ± 0.041533	0.97	−0.030477 ± 0.170373	0.85	−0.006164 ± 0.058302	0.92	0.047106 ± 0.04965	0.34	−0.111058 ± 0.075964	0.14
INTREF	−0.03068 ± 0.013019	0.018	−0.08130 ± 0.065117	0.21	0.029161 ± 0.019725	0.14	−0.044635 ± 0.01416	0.002	0.017892 ± 0.030217	0.55
INTMED	−0.002518 ± 0.001091	0.021	−0.014963 ± 0.012300	0.22	−0.002209 ± 0.0015200	0.15	−0.004101 ± 0.001370	0.003	0.001156 ± 0.001961	0.56
PIE	0.025258 ± 0.00825	0.002	*0.056378 ± 0.030734*	*0.067*	*0.020223 ± 0.010543*	*0.055*	0.035731 ± 0.01014	<0.001	−0.000623 ± 0.015240	0.97
HORVATH 2 DNAmage										
TE	0.008776 ± 0.023368	0.71	0.144643 ± 0.09645	0.13	0.005491 ± 0.031364	0.86	0.044246 ± 0.028728	0.12	*−0.066184 ± 0.039959*	*0.098*
CDE	0.007917 ± 0.027017	0.77	0.1593 ± 0.114640	0.17	0.003622 ± 0.037059	0.92	0.04823 ± 0.0324588	0.14	*−0.088714 ± 0.049106*	*0.071*
INTREF	−0.001772 ± 0.008428	0.83	−0.026870 ± 0.043601	0.54	0.004768 ± 0.012505	0.70	−0.009105 ± 0.009134	0.32	0.02695 ± 0.019595	0.17
INTMED	−0.000145 ± 0.000692	0.83	−0.004946 ± 0.008080	0.54	0.00036 ± 0.000948	0.70	−0.000837 ± 0.000844	0.32	0.00174 ± 0.001296	0.18
PIE	0.00278 ± 0.005128	0.59	0.017188 ± 0.019522	0.38	−0.003260 ± 0.006489	0.62	0.005958 ± 0.006068	0.33	−0.006164 ± 0.009900	0.53
LIN DNAmage										
TE	−0.00242 ± 0.040121	0.95	−0.084943 ± 0.157876	0.59	0.039006 ± 0.055693	0.48	0.03899 ± 0.049803	0.43	−0.087385 ± 0.066179	0.19
CDE	−0.013629 ± 0.046383	0.77	−0.037610 ± 0.18775	0.84	0.020999 ± 0.065761	0.75	0.03768 ± 0.056255	0.50	*−0.137368 ± 0.081327*	*0.091*
INTREF	0.002047 ± 0.014474	0.89	−0.033086 ± 0.071486	0.64	0.000245 ± 0.022189	0.99	−0.012547 ± 0.015824	0.43	0.052325 ± 0.032490	0.11
INTMED	0.00017 ± 0.001188	0.89	−0.006087 ± 0.013209	0.65	0.000019 ± 0.001681	0.99	−0.001153 ± 0.00146	0.43	0.003382 ± 0.002167	0.12
PIE	0.008998 ± 0.008837	0.31	−0.008161 ± 0.031576	0.80	0.017743 ± 0.011735	0.13	0.01501 ± 0.01060	0.16	−0.005723 ± 0.016340	0.73
WEIDNER DNAmage										
TE	−0.075615 ± 0.056708	0.18	0.072339 ± 0.225908	0.75	−0.078087 ± 0.078398	0.32	−0.087536 ± 0.069939	0.21	−0.04964 ± 0.09565	0.60
CDE	*−0.115592 ± 0.065554*	*0.078*	0.138139 ± 0.267984	0.61	*−0.153137 ± 0.092570*	*0.098*	−0.103996 ± 0.079041	0.19	−0.155799 ± 0.117465	0.19
INTREF	0.024971 ± 0.020475	0.22	−0.11638 ± 0.10225	0.26	0.070229 ± 0.031396	0.025	−0.000603 ± 0.022202	0.98	0.09694 ± 0.047073	0.039
INTMED	0.002050 ± 0.001690	0.23	−0.021425 ± 0.01924	0.27	0.005320 ± 0.002468	0.031	−0.000055 ± 0.002040	0.98	*0.00627 ± 0.003198*	*0.050*
PIE	0.012956 ± 0.012494	0.30	0.07200 ± 0.04719	0.13	−0.000497 ± 0.016164	0.98	0.017118 ± 0.014798	0.25	0.002958 ± 0.023611	0.90
VIDAL−BRALO DNAmage										
TE	−0.019887 ± 0.026611	0.46	−0.027050 ± 0.106646	0.80	−0.003859 ± 0.036556	0.92	−0.014628 ± 0.03288	0.66	−0.026090 ± 0.044507	0.56
CDE	−0.027454 ± 0.030747	0.37	0.030208 ± 0.126501	0.81	−0.02834 ± 0.04319	0.51	−0.010470 ± 0.037116	0.78	−0.069110 ± 0.054711	0.21
INTREF	−0.001778 ± 0.009594	0.85	−0.074933 ± 0.048398	0.12	0.020630 ± 0.014605	0.16	−0.013839 ± 0.0104560	0.19	*0.036938 ± 0.021874*	*0.091*
INTMED	−0.00015 ± 0.000788	0.85	−0.013794 ± 0.00927	0.14	0.001562 ± 0.00112	0.16	−0.001272 ± 0.00010	0.19	0.002387 ± 0.001463	0.10
PIE	0.009491 ± 0.005903	0.11	0.031469 ± 0.022131	0.16	0.002283 ± 0.007554	0.76	0.010952 ± 0.007011	0.12	0.003696 ± 0.011004	0.74
YANG DNAmage										
TE	−0.000109 ± 0.000010	0.26	−0.000399 ± 0.000421	0.34	−0.000154 ± 0.000123	0.21	−0.000282 ± 0.00012	0.012	0.00026 ± 0.000167	0.12
CDE	−0.000137 ± 0.000111	0.22	−0.000386 ± 0.000500	0.44	−0.000212 ± 0.000145	0.15	−0.000297 ± 0.000132	0.025	0.000213 ± 0.000205	0.30
INTREF	0.000024 ± 0.000035	0.50	−0.000027 ± 0.000191	0.89	0.000069 ± 0.000049	0.16	0.000010 ± 0.000037	0.78	0.000038 ± 0.000082	0.64
INTMED	1.93e−06 ± 2.85e−06	0.50	−5.00e−06 ± 0.000035	0.89	5.25e−06 ± 3.77e−06	0.16	9.35e−07 ± 3.42e−06	0.78	2.47e−06 ± 5.29e−06	0.64
PIE	2.29e−06 ± 0.000021	0.91	0.000019 ± 0.000084	0.83	−0.000017 ± 0.000025	0.51	4.07e−06 ± 0.000025	0.87	4.90e−06 ± 0.000041	0.91
ZHANG DNAmage										
TE	−0.003287 ± 0.002114	0.12	0.009301 ± 0.008632	0.28	−0.0056307 ± 0.002862	0.049	−0.003429 ± 0.002585	0.19	−0.003088 ± 0.003660	0.40
CDE	−0.003529 ± 0.002443	0.15	0.015163 ± 0.010208	0.14	*−0.0059164 ± 0.0033824*	*0.080*	−0.003914 ± 0.002923	0.18	−0.002639 ± 0.004502	0.56
INTREF	−0.000226 ± 0.000762	0.77	−0.0085045 ± 0.003926	0.030	0.0001439 ± 0.0011408	0.90	−0.0000836 ± 0.00082	0.92	−0.000790 ± 0.001790	0.66
INTMED	−0.000019 ± 0.000063	0.77	−0.001566 ± 0.000779	0.044	0.0000109 ± 0.0000864	0.90	−7.68e−06 ± 0.0000753	0.92	−0.000051 ± 0.000116	0.66
PIE	0.0004862 ± 0.0004667	0.30	0.004208 ± 0.00191	0.028	0.0001309 ± 0.0005916	0.83	0.000576 ± 0.0005458	0.29	0.000392 ± 0.000908	0.67
BOCKLANDT DNAmage										
TE	*0.000668 ± 0.000373*	*0.073*	*0.002839 ± 0.001508*	*0.060*	0.0002343 ± 0.0005079	0.65	0.000313 ± 0.000439	0.48	*0.001368 ± 0.000708*	*0.053*
CDE	0.000661 ± 0.000431	0.13	0.001869 ± 0.001789	0.30	0.0002495 ± 0.0006001	0.68	0.000218 ± 0.000496	0.66	*0.001681 ± 0.000871*	*0.054*
INTREF	0.000068 ± 0.000134	0.61	*0.001144 ± 0.000686*	*0.096*	0.0000331 ± 0.0002025	0.87	0.000167 ± 0.000140	0.23	−0.0003 ± 0.0003	0.39
INTMED	5.60e−06 ± 0.00001	0.61	0.000211 ± 0.000132	0.11	2.51e−06 ± 0.0000153	0.87	0.000015 ± 0.000013	0.23	−0.000019 ± 0.000023	0.39
PIE	−0.000067 ± 0.000082	0.41	−0.000384 ± 0.000311	0.22	−0.0000508 ± 0.0001051	0.63	−0.000088 ± 0.000093	0.34	6.60e−06 ± 0.000175	0.97
GARAGNANI DNAmage										
TE	0.000163 ± 0.000288	0.57	0.001057 ± 0.001152	0.36	0.000136 ± 0.000396	0.73	0.000017 ± 0.000358	0.96	0.000619 ± 0.000471	0.19
CDE	0.000079 ± 0.000333	0.81	0.001236 ± 0.001368	0.37	5.27e−06 ± 0.000468	0.99	7.10e−07 ± 0.000404	1.00	0.0004 ± 0.000580	0.49
INTREF	0.000048 ± 0.000104	0.65	−0.000388 ± 0.000521	0.46	0.000130 ± 0.00016	0.41	8.96e−06 ± 0.000114	0.94	0.000092 ± 0.000230	0.69
INTMED	3.90e−06 ± 8.53e−06	0.65	−0.000071 ± 0.000097	0.46	9.80e−06 ± 0.000012	0.42	8.24e−07 ± 0.000010	0.94	5.95e−06 ± 0.000015	0.69
PIE	0.000033 ± 0.000063	0.60	0.000281 ± 0.000237	0.24	−8.95e−06 ± 0.000082	0.91	6.31e−06 ± 0.000075	0.93	0.0001208 ± 0.00012	0.31
DNAm GRIMAGE										
TE	−0.027040 ± 0.019139	0.16	−0.001467 ± 0.081877	0.99	−0.012447 ± 0.02532	0.62	−0.03164 ± 0.023365	0.18	−0.019421 ± 0.033951	0.57
CDE	−0.033468 ± 0.022122	0.13	−0.0040010 ± 0.097353	0.97	−0.011468 ± 0.0299	0.70	−0.039733 ± 0.026396	0.13	−0.020376 ± 0.041777	0.63
INTREF	0.001924 ± 0.006879	0.78	−0.020173 ± 0.0364	0.58	−0.004709 ± 0.010090	0.64	0.002957 ± 0.007331	0.69	−0.000380 ± 0.016601	0.98
INTMED	0.000158 ± 0.000565	0.78	−0.003714 ± 0.00674	0.58	−0.000357 ± 0.000766	0.64	0.000272 ± 0.000674	0.69	−0.000025 ± 0.001073	0.98
PIE	0.004346 ± 0.004228	0.30	0.026429 ± 0.016834	0.12	0.004086 ± 0.005269	0.44	0.004862 ± 0.00488	0.32	0.0014 ± 0.00840	0.87
MPOA										
TE	−0.000075 ± 0.000430	0.86	0.001808 ± 0.001812	0.32	−0.000360 ± 0.000569	0.53	−0.000144 ± 0.000524	0.78	0.000037 ± 0.000755	0.96
CDE	0.000073 ± 0.000496	0.88	0.002254 ± 0.00215	0.30	−0.000047 ± 0.000672	0.95	0.000024 ± 0.000592	0.97	0.000022 ± 0.000930	0.98
INTREF	−0.000201 ± 0.000155	0.19	−0.000619 ± 0.000816	0.45	−0.000367 ± 0.000227	0.11	−0.000234 ± 0.000166	0.16	0.0000330 ± 0.000370	0.93
INTMED	−0.000017 ± 0.000013	0.20	−0.000114 ± 0.000152	0.45	−0.000028 ± 0.000018	0.11	−0.000022 ± 0.000015	0.16	2.11e−06 ± 0.000024	0.93
PIE	0.000069 ± 0.000095	0.47	0.000288 ± 0.000364	0.43	0.00008 ± 0.000118	0.49	0.000087 ± 0.000110	0.43	−0.000020 ± 0.00019	0.92

Abbreviations: CDE: Controlled Direct Effect; CES-D: Centers for Epidemiological Studies-Depression; DNAm: DNA methylation; DNAmage: DNA methylation age; INTMED: Mediated Interaction; INTREF: Interaction referent; PIE: Pure Indirect Effect; TE: Total Effect. See Supplementary Methods for epigenetic clock abbreviations. ^a^OLS regression models with epigenetic clocks as alternative outcomes and point EOD measured in 2010–2012 as exposures and CES-D scores measured in 2014–2016 as a potential mediator, sample size *N* = 2,806, four-way mediation analysis. Stratified analysis by sex and/or race was also presented. Italicized findings have *p* < 0.10 but >0.05. ^b^Exogenous variables are the ones included in Table 2, Model 3, as covariates. See Covariates section for detail.

**Table 4:** Reasons for Perceived discrimination (RPD: 2010/2012) → depressive symptoms (CES-D: 2014/2016) → epigenetic clocks (2016): 4-way mediation analysis, overall and by sex and race, full model: HRS 2010-2016.

**Y = Epigenetic clock**	**Overall**		**Males**		**Females**		**NHW**		**Non-Whites**	
	**β ± SE**	***P***	**β ± SE**	***P***	**β ± SE**	***P***	**β ± SE**	***P***	**β ± SE**	***P***
HORVATH DNAmage										
TE	−0.1032053 ± 0.090445	0.25	−0.1878292 ± 0.1379343	0.17	−0.0606206 ± 0.119885	0.61	−0.0663948 ± 0.1246099	0.59	−0.104212 ± 0.130395	0.42
CDE	−0.1180829 ± 0.1079943	0.27	−0.2006204 ± 0.1636529	0.22	−0.0738707 ± 0.1451321	0.61	−0.0581158 ± 0.1457803	0.69	−0.1710287 ± 0.1646686	0.30
INTREF	0.026679 ± 0.0394156	0.50	0.0475597 ± 0.0623305	0.45	0.0187913 ± 0.0531213	0.72	0.009112 ± 0.0462641	0.84	0.0725672 ± 0.0828621	0.38
INTMED	0.0046929 ± 0.0069552	0.50	0.008759 ± 0.011595	0.45	0.0032557 ± 0.009223	0.72	0.0024243 ± 0.0123143	0.84	0.0053665 ± 0.0063799	0.40
PIE	−0.0164942 ± 0.0174394	0.34	−0.0435274 ± 0.0287969	0.13	−0.0087971 ± 0.0231059	0.70	−0.0198152 ± 0.0273385	0.47	−0.0111174 ± 0.0190018	0.56
HANNUM DNAmage										
TE	−0.02898 ± 0.0725739	0.69	−0.0101855 ± 0.1113979	0.93	−0.0386383 ± 0.0954672	0.69	0.0645212 ± 0.1004553	0.52	−0.143671 ± 0.1017024	0.16
CDE	−0.014727 ± 0.0866313	0.87	0.019218 ± 0.1324281	0.89	−0.0300982 ± 0.1154603	0.79	0.0933474 ± 0.1174426	0.43	−0.161799 ± 0.1286189	0.21
INTREF	−0.0339282 ± 0.0316215	0.28	−0.0409591 ± 0.0503832	0.42	−0.0284703 ± 0.0422382	0.50	−0.0523597 ± 0.0373693	0.16	0.0059789 ± 0.064886	0.93
INTMED	−0.0059679 ± 0.005605	0.29	−0.007538 ± 0.0093789	0.42	−0.0049375 ± 0.007361	0.50	−0.0139327 ± 0.0100693	0.17	0.0004443 ± 0.0048012	0.93
PIE	*0.0256432 ± 0.0142343*	*0.072*	0.0190936 ± 0.0225292	0.40	0.0248677 ± 0.0187434	0.19	*0.0374663 ± 0.0224163*	*0.095*	0.0117047 ± 0.0150756	0.44
LEVINE DNAmage										
TE	−0.0324966 ± 0.0951323	0.73	−0.0703583 ± 0.1437414	0.63	0.0112261 ± 0.1266309	0.93	0.1753391 ± 0.1310727	0.18	−*0.2572829 ± 0.1352268*	*0.057*
CDE	−0.0020692 ± 0.1134302	0.99	−0.0304774 ± 0.1703734	0.86	0.0466778 ± 0.153067	0.76	0.2191643 ± 0.1528806	0.15	−0.2636591 ± 0.1710249	0.12
INTREF	−0.0659782 ± 0.0414743	0.11	−0.081296 ± 0.0651171	0.21	−0.0645208 ± 0.0560374	0.25	−0.1019176 ± 0.0488054	0.037	0.0014963 ± 0.0864472	0.99
INTMED	−0.0116065 ± 0.0074194	0.12	−0.0149631 ± 0.0122998	0.22	−0.0111899 ± 0.0098586	0.26	−0.0271207 ± 0.01335	0.042	0.0001103 ± 0.0063933	0.99
PIE	0.0471573 ± 0.0191017	0.014	*0.0563782 ± 0.0307344*	*0.067*	0.040259 ± 0.0251081	0.11	0.0852131 ± 0.0304577	0.005	0.0047695 ± 0.019479	0.81
HORVATH 2 DNAmage										
TE	0.053499 ± 0.06181	0.39	0.144643 ± 0.09645	0.13	−0.020860 ± 0.080319	0.80	0.1987906 ± 0.0852737	0.020	−0.107497 ± 0.087803	0.22
CDE	0.067841 ± 0.073822	0.36	0.1593 ± 0.114640	0.17	−0.012433 ± 0.097216	0.90	0.228888 ± 0.099735	0.022	−0.141781 ± 0.111056	0.20
INTREF	−0.021002 ± 0.026917	0.44	−0.026870 ± 0.043601	0.54	−0.006618 ± 0.035542	0.85	−0.03972 ± 0.031701	0.21	0.035025 ± 0.05612	0.53
INTMED	−0.003695 ± 0.004755	0.44	−0.004946 ± 0.008080	0.54	−0.001148 ± 0.00617	0.85	−0.010570 ± 0.008521	0.22	0.002590 ± 0.004237	0.54
PIE	0.010354 ± 0.011893	0.38	0.017188 ± 0.019522	0.38	−0.000662 ± 0.015445	0.97	0.020193 ± 0.018780	0.22	−0.003331 ± 0.012621	0.79
LIN DNAmage										
TE	−0.0418754 ± 0.1060137	0.69	−0.084943 ± 0.157876	0.59	−0.011092 ± 0.142675	0.94	0.172602 ± 0.147802	0.24	−0.227671 ± 0.146110	0.12
CDE	−0.0594891 ± 0.1266206	0.64	−0.0376095 ± 0.187751	0.84	−0.061277 ± 0.172529	0.72	0.200682 ± 0.172832	0.25	*−0.339307 ± 0.184817*	*0.066*
INTREF	−0.0060369 ± 0.0461974	0.90	−0.033086 ± 0.071486	0.64	0.006160 ± 0.063100	0.92	−0.063740 ± 0.054962	0.25	0.1057601 ± 0.0931083	0.26
INTMED	−0.001062 ± 0.0081282	0.90	−0.006087 ± 0.013209	0.65	0.001070 ± 0.010943	0.92	−0.016962 ± 0.014754	0.25	0.00782 ± 0.00735	0.29
PIE	0.0247126 ± 0.0205325	0.23	−0.008161 ± 0.031576	0.80	0.042959 ± 0.028215	0.13	0.052622 ± 0.032936	0.11	−0.00194 ± 0.020850	0.93
WEIDNER DNAmage										
TE	−0.0753708 ± 0.1498217	0.62	0.0723385 ± 0.2259075	0.75	−0.170970 ± 0.200762	0.39	−0.144141 ± 0.207537	0.49	0.0183725 ± 0.2099748	0.93
CDE	−0.1540839 ± 0.178938	0.39	0.1381392 ± 0.2679837	0.61	−0.331622 ± 0.242913	0.17	−0.163069 ± 0.242820	0.50	−0.1703086 ± 0.265165	0.52
INTREF	0.0347483 ± 0.0653234	0.60	−0.11638 ± 0.1022509	0.26	0.119809 ± 0.088989	0.18	−0.031192 ± 0.077091	0.69	0.1572077 ± 0.1343571	0.24
INTMED	0.0061148 ± 0.0115156	0.60	−0.0214248 ± 0.01924	0.27	0.020777 ± 0.01573	0.19	−0.008300 ± 0.02054	0.69	0.0116254 ± 0.0106502	0.28
PIE	0.03785 ± 0.029085	0.19	0.0720041 ± 0.0471927	0.13	0.020066 ± 0.038718	0.60	0.058420 ± 0.045904	0.20	0.0198481 ± 0.0308601	0.52
VIDAL−BRALO DNAmage										
TE	0.0603942 ± 0.070296	0.39	−0.027050 ± 0.106646	0.80	0.1358484 ± 0.0936072	0.15	0.09527 ± 0.097587	0.33	0.050888 ± 0.097809	0.60
CDE	0.0514491 ± 0.0839531	0.54	0.030208 ± 0.126501	0.81	0.1021091 ± 0.1132749	0.37	0.124348 ± 0.114138	0.28	−0.025604 ± 0.123661	0.84
INTREF	−0.0085175 ± 0.0306371	0.78	−0.074933 ± 0.048398	0.12	0.0170052 ± 0.041415	0.68	−0.044923 ± 0.036295	0.22	0.062517 ± 0.062576	0.32
INTMED	−0.0014978 ± 0.0053924	0.78	−0.013794 ± 0.00927	0.14	0.0029485 ± 0.0071947	0.68	−0.011955 ± 0.009755	0.22	0.004623 ± 0.004873	0.34
PIE	0.0189605 ± 0.0136641	0.17	0.031469 ± 0.022131	0.16	0.0137857 ± 0.0181255	0.45	0.027803 ± 0.021586	0.20	0.009352 ± 0.014363	0.52
YANG DNAmage										
TE	−0.0003615 ± 0.0002536	0.15	−0.000399 ± 0.000421	0.34	−0.000292 ± 0.000315	0.35	−0.000904 ± 0.000347	0.009	0.00030 ± 0.000370	0.42
CDE	*−0.0005061 ± 0.000303*	*0.095*	−0.000386 ± 0.000500	0.44	−0.000478 ± 0.000381	0.21	−0.000904 ± 0.000406	0.026	−0.000190 ± 0.000462	0.68
INTREF	0.0001347 ± 0.0001107	0.22	−0.000027 ± 0.00019	0.89	0.000195 ± 0.000139	0.16	−0.000029 ± 0.000129	0.82	0.000488 ± 0.000236	0.038
INTMED	0.0000237 ± 0.0000197	0.23	−5.00e−06 ± 0.0000351	0.89	0.000034 ± 0.000025	0.17	−7.76e−06 ± 0.000034	0.82	*0.000036 ± 0.000021*	*0.087*
PIE	−0.0000139 ± 0.0000486	0.73	0.0000186 ± 0.0000843	0.83	−0.000044 ± 0.000061	0.48	0.000037 ± 0.000076	0.62	−0.000039 ± 0.00005	0.47
ZHANG DNAmage										
TE	−0.0002539 ± 0.0055824	0.96	0.0093006 ± 0.0086322	0.28	−0.006003 ± 0.007335	0.39	0.000849 ± 0.007667	0.91	−0.000198 ± 0.008032	0.98
CDE	0.0007082 ± 0.0066688	0.92	0.0151633 ± 0.0102084	0.14	−0.007674 ± 0.008879	0.39	0.001933 ± 0.008970	0.83	0.000814 ± 0.01017	0.94
INTREF	−0.0017867 ± 0.0024345	0.46	−0.0085048 ± 0.0039263	0.030	0.001753 ± 0.003247	0.59	−0.002714 ± 0.002848	0.34	−0.001045 ± 0.005136	0.84
INTMED	−0.0003143 ± 0.0004298	0.47	−0.0015657 ± 0.0007789	0.044	0.000304 ± 0.000565	0.59	−0.000722 ± 0.000762	0.34	−0.000077 ± 0.000381	0.84
PIE	0.0011389 ± 0.0010811	0.29	0.0042079 ± 0.001909	0.028	−0.000387 ± 0.001414	0.79	0.002353 ± 0.001701	0.17	0.0001096 ± 0.001153	0.92
BOCKLANDT DNAmage										
TE	0.001002 ± 0.000984	0.31	*0.002839 ± 0.001508*	*0.060*	−0.00053 ± 0.001301	0.68	−0.001001 ± 0.00130	0.44	*0.002978 ± 0.001555*	*0.056*
CDE	0.00099 ± 0.001176	0.40	0.001869 ± 0.001789	0.30	−0.000325 ± 0.001574	0.84	−0.001407 ± 0.001525	0.36	0.003883 ± 0.00197	0.048
INTREF	0.000072 ± 0.000429	0.87	*0.001144 ± 0.000686*	*0.096*	−0.000165 ± 0.000576	0.77	0.000440 ± 0.000485	0.36	−0.000891 ± 0.000995	0.37
INTMED	0.000013 ± 0.000076	0.87	0.000211 ± 0.000132	0.11	−0.000029 ± 0.000100	0.77	0.000117 ± 0.000130	0.37	−0.000066 ± 0.000077	0.39
PIE	−0.000073 ± 0.000189	0.70	−0.000384 ± 0.000311	0.22	−9.56e−06 ± 0.000250	0.97	−0.000152 ± 0.000285	0.59	0.000052 ± 0.000224	0.82
GARAGNANI DNAmage										
TE	0.000126 ± 0.000760	0.87	0.001057 ± 0.001152	0.36	−0.000416 ± 0.001014	0.68	0.001075 ± 0.001061	0.31	−0.000775 ± 0.001034	0.45
CDE	−0.000113 ± 0.000908	0.90	0.001236 ± 0.001368	0.37	−0.000882 ± 0.001227	0.47	0.001303 ± 0.001242	0.29	−0.001721 ± 0.001307	0.19
INTREF	0.000125 ± 0.000332	0.91	−0.000388 ± 0.000521	0.46	0.000409 ± 0.000449	0.36	−0.000268 ± 0.000395	0.50	0.000772 ± 0.000662	0.24
INTMED	0.00002 ± 0.000058	0.71	−0.000071 ± 0.000097	0.46	0.000071 ± 0.000079	0.37	−0.000071 ± 0.000105	0.50	0.000057 ± 0.000053	0.28
PIE	0.000092 ± 0.000146	0.52	0.0002808 ± 0.0002365	0.24	−0.000013 ± 0.00020	0.95	0.000112 ± 0.000232	0.63	0.000117 ± 0.000153	0.45
DNAm GRIMAGE										
TE	*0.087878 ± 0.050904*	*0.084*	−0.001467 ± 0.081877	0.99	0.171512 ± 0.064623	0.008	0.033214 ± 0.0688172	0.63	*0.141369 ± 0.075061*	*0.060*
CDE	0.088810 ± 0.06089	0.15	−0.004009 ± 0.097353	0.97	0.189456 ± 0.078228	0.015	0.0134562 ± 0.0804868	0.87	*0.186614 ± 0.095129*	*0.050*
INTREF	−0.009309 ± 0.021986	0.67	−0.020173 ± 0.0364	0.56	−0.020831 ± 0.028617	0.47	0.0065834 ± 0.0254117	0.80	−0.047560 ± 0.04769	0.32
INTMED	−0.001639 ± 0.003873	0.67	−0.003714 ± 0.00674	0.58	−0.003614 ± 0.004993	0.47	0.0017513 ± 0.0067651	0.80	−0.003515 ± 0.003711	0.34
PIE	0.010017 ± 0.009774	0.31	0.026429 ± 0.016834	0.12	0.006501 ± 0.012530	0.60	0.011423 ± 0.0150537	0.45	0.005830 ± 0.010831	0.59
MPOA										
TE	*0.002094 ± 0.001137*	*0.065*	0.001808 ± 0.001812	0.32	0.002363 ± 0.00146	0.11	0.001748 ± 0.001546	0.26	0.002576 ± 0.001665	0.12
CDE	0.002927 ± 0.001359	0.031	0.002254 ± 0.00215	0.30	0.003483 ± 0.001762	0.048	0.002248 ± 0.001809	0.21	0.004332 ± 0.002102	0.039
INTREF	−0.000844 ± 0.000495	0.088	−0.000619 ± 0.000816	0.45	−0.001044 ± 0.000646	0.11	−0.000435 ± 0.0005743	0.45	*−0.001860 ± 0.001064*	*0.080*
INTMED	−0.000149 ± 0.000089	0.094	−0.000114 ± 0.000152	0.45	−0.000181 ± 0.000115	0.12	−0.000116 ± 0.000153	0.45	−0.000138 ± 0.000091	0.13
PIE	0.000160 ± 0.000219	0.46	0.000288 ± 0.000364	0.43	0.000106 ± 0.000282	0.71	0.000050 ± 0.000338	0.88	0.000242 ± 0.000251	0.33

Abbreviations: CDE: Controlled Direct Effect; CES-D: Centers for Epidemiological Studies-Depression; DNAm: DNA methylation; DNAmage: DNA methylation age; INTMED=Mediated Interaction; INTREF=Interaction referent; PIE: Pure Indirect Effect; TE: Total Effect. See Supplementary Methods for epigenetic clock abbreviations. ^a^OLS regression models with epigenetic clocks as alternative outcomes and point RPD measured in 2010–2012 as exposures and CES-D scores measured in 2014–2016 as a potential mediator, sample size *N* = 2,806, four-way mediation analysis. Stratified analysis by sex and/or race was also presented. Italicized findings have *p* < 0.10 but >0.05. ^b^Exogenous variables are the ones included in Table 2, Model 3, as covariates. See Covariates section for detail.

**Figure 3:**
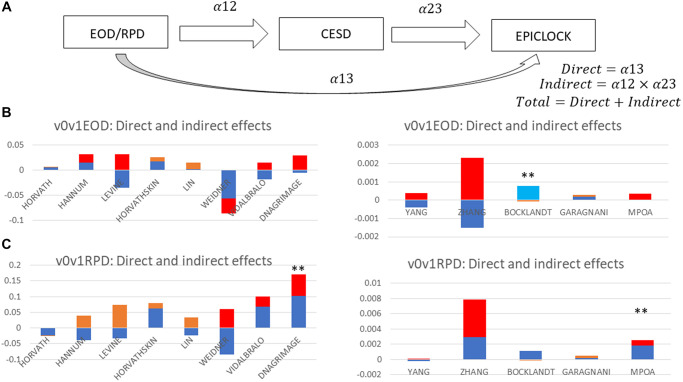
**Total, direct and indirect effects of perceived discrimination measures on epigenetic clocks through depressive symptoms: structural equations modeling (sem); hrs 2010–2016 (*n* = 2,806).** (**A**) SEM mediation model. (**B**) EOD as Exposure. (**C**) RPD as Exposure. Abbreviations: EOD: Experience of Discrimination; HRS: Health and Retirement Study; RPD: Reasons for perceived discrimination; v0: baseline visit, wave 10 (2010); v1: first follow-up visit, wave 11 (2012); v2: second follow-up visit, wave 12 (2014); v3: third follow-up visit, wave 13 (2016); v0v1: combined visits 0 and 1; v2v3: combined visits 2 and 3. See Supplementary Methods for epigenetic clock abbreviations. Note 1: SEM was conducted on epigenetic clocks as alternative outcomes, v2v3CESD total score as the mediator and EOD or RPD at v0v1 as alternative exposures. Exogenous variables are estimated age at v3, sex, and race. Figure 3 decomposes the total into direct and indirect effects of each measure of perceived discrimination. All path coefficients are shown in detail in Supplementary Table 5. Note 2: Red = Significant indirect effect at type I error of 0.05; Light blue = Significant direct effect at type I error of 0.05; Orange = Indirect effect, *P* > 0.05 for null hypothesis indirect effect = 0; Dark blue = Direct effect, *P* > 0.05 for null hypothesis direct effect = 0; ^**^P < 0.05 for null hypothesis total effect = 0.

In our SEM sensitivity analyses (Supplementary Table 5 and Figure 3), we found that overall, and based on a minimally adjusted model, the total effect of EOD on the epigenetic clocks was largely null with the exception of the BOCKLANDT clock which indicated a positive and largely direct effect, reflecting greater biological age with perceive discrimination that is not mediated through CES-D total score. In contrast, for RPD, the total effect was statistically significant in the case of MPOA and DNAGrimage, which was significantly mediated through CES-D total score. These latter findings for MPOA and DNAGrimage are in line with the previous analyses in Supplementary Table 4.

## DISCUSSION

Here we examined retrospective data from the HRS cohort study of U.S. adults aged 50–100 years and investigated measures of perceived discrimination and depressive symptoms in relation to 13 different DNAm-based epigenetic clocks (EPICLOCK) age-estimators. We applied two distinct methodologies and utilized 4-way mediation analyses to decompose total effects of perceived discrimination markers on epigenetic age through depressive symptoms, in a time-dependent manner between 2010 and 2016. We also evaluated whether these associations differed by sex and race/ethnicity groups, separately. In the overall cohort and mostly among female and NHW participants, a higher RPD had a significant adverse total effect on short term (~2–6 yrs) epigenetic aging, based on the DNAm GrimAge, MPOA, Levine (PhenoAge) and Horvath 2 epigenetic clocks. This effect could partially (20–50%) be explained by a pure indirect effect through depressive symptoms. These total effects were not detected in either model for EOD. Among females, elevated DEP was associated with faster epigenetic aging in the LIN clock in the reduced model, with an attenuation in subsequent models, patterns observed for elevated DEP (high vs. low) for GrimAge and MPOA DNAm markers. Overall and in White adults, EOD/RPD-Levine clock relationship was purely explained by variations in the CES-D total score, or DEP.

MDD and depressive symptoms have been both linked to epigenetic aging or age acceleration. For instance, using data from the Netherlands Study of Depression and Anxiety (NESDA), Han et al. reported significantly higher epigenetic aging in patients with MDD (*n* = 319) compared to controls (*N* = 811), using a cutoff of 14 for the Inventory of Depressive Symptomology with a follow up of 4 years [17]. Nevertheless, their findings also suggested that faster epigenetic aging in MDD may be largely explained by illness severity, although no additional relationships were uncovered between higher epigenetic aging and cumulative clinical characteristics [17]. This study also used a different technology, MBD-Seq, for DNAm measurements and therefore a unique DNAm age estimator was utilized to accommodate using DNAm data from MBD-Seq versus the more standard Illumina technology [17]. Our recent analysis of the Healthy Aging of Neighborhoods of Diversity Across the Life Span (HANDLS) study detected a cross-sectional association between two epigenetic age acceleration measures (using Horvath 1 and Hannum clocks) and lower positive affect among White adults. Despite a lack of association between these epigenetic clocks and race, White adults compared with African American adults had a lower positive affect, even upon adjustment for age, sex, and poverty status (*P* = 0.007), suggesting that White urban adults may be more affected by epigenetic age acceleration due to their reduced level of positive affect at baseline [6]. In a recent case-control study of 49 MDD cases and age-matched controls (*n* = 60), MDD was associated with GrimAge acceleration [16]. GrimAge is a recently developed second generation epigenetic clock that is a composite clock that includes proxy DNAm biomarkers of 7 different plasma proteins and a DNAm-based estimator of smoking pack-years [48]. In this study the relationship between GrimAge and MDD remained significant upon adjustment for sex, current smoking status and BMI (*p* = 0.015) [16]. In our study, GrimAge DNAm age was associated with sustained elevated depressive symptoms over time. Nevertheless, this association was markedly attenuated with adjustment for smoking and BMI among lifestyle and health-related factors. This is consistent with our previous study with HANDLS data [6] in which we found only a partial association with the “positive affect” domain of depression and no association with depressive symptoms and epigenetic aging using the Horvath and Hannum epigenetic clocks.

Several studies have indicated that perceived ethnic or racial discrimination were associated with elevated depressive symptoms, with little evidence of a buffering effect of coping mechanisms among Black men [49]. While our study did not focus on racial/ethnic or gender perceived discrimination per se, the number of reasons for perceived discrimination was greater among women and among racial/ethnic minorities. Thus, RPD is expected to be in part reflecting perceptions of gender and/or racial/ethnic discrimination. Pure indirect effects detected in our study suggest that RPD and/or EOD are positively linked to depressive symptoms which in turn results in epigenetic age acceleration. Thus, potentially manipulating RPD may lower epigenetic age mainly through the reduction of depressive symptoms. This was specifically the case for the MPOA and DNAGrimAge clocks, in the overall sample, according to our sensitivity analysis using SEM models, consistently with our 4-way mediation analyses. In contrast, the EOD was found to have a positive total effect on the BOCKLANDT clock, which was a direct one. Given the nature of the BOCKLANDT clock, which is shown to be inversely related to the remaining 12 clocks, this result indicates that EOD may be protective against epigenetic age acceleration, an effect that is not explained by depressive symptoms and was specifically detected among Non-White adults. In addition, this result was not consistent with our 4-way mediation analysis. Nevertheless, this finding needs to be replicated in other comparable cohorts in order to come to a firm conclusion.

To our knowledge, our study is among a few to systematically examine perceived discrimination in relation to epigenetic aging. In African American mothers, major life discrimination, but not race-related events measures, were associated with DNAm at 9 different CpG sites [50]. However, in this study epigenetic age parameters were not assessed. Using data on low-income middle-aged Black adults (*n* = 219), a recent study detected an association of race-related lifetime stress exposure (i.e., exposure to racial discrimination, trauma, and stressful life events) and epigenetic age acceleration that was fully mediated through internalized and externalized anger expression [51]. Our present study indicated that in fact, perceived discrimination has little potential direct effect on the epigenetic clocks with few exceptions, and that its total effect when present is strongly mediated through depressive symptoms in at least 4 of 13 epigenetic clocks considered (DNAm GrimAge, MPOA, Levine (PhenoAge) and Horvath 2), particularly among females and NHW. Moreover, similar to the study by McKenna et al. [51], non-significant total effects for some of the epigenetic clocks were accompanied by a pure indirect effect through depressive symptoms, particularly in reduced models, that were only adjusted for basic demographics. Typically, but not consistently, results from our analyses indicate a stronger, negative association between perceived discrimination/reasons for perceived discrimination, depressive symptoms, and epigenetic clocks among NHW relative to Non-Whites. Moreover, compared to men, the associations between perceived discrimination, reasons for perceived discrimination, depressive symptoms, and epigenetic clocks among women tended to be stronger and positive. These findings are supported by the extant literature in which researchers have reported discrepant associations between various SES and health inputs to outcomes among gender and racial/ethnic groups. For example, researchers have documented lower health returns to markers of SES—such as educational attainment—among non-white relative to white adults in the US [52–55].

Although there are few studies that have examined the effects of perceived discrimination on epigenetic mechanisms, more studies have examined the effects of other psychosocial stressors on epigenetic aging (reviewed in [56]). Several studies point to the association of socioeconomic status, trauma and lifetime stress with accelerated epigenetic aging [56]. However, there are many discordant findings in the field, which may reflect the complex nature of analyzing psychosocial stressors. These effects may depend upon the nature of the stressor (acute versus chronic), different sociodemographic makeup of the cohorts and various other parameters. Nevertheless, as exposure to psychosocial stressors negatively impact health outcomes [18], alterations in epigenetic mechanisms provide a pathway by which these stressors may influence health. In line with this idea, one third of the DNAm sites in the Horvath epigenetic clock are localized within glucocorticoid response elements [57]. Glucocorticoids released in response to stress can then potentially modify DNAm and affect epigenetic age [57]. Most recently, data suggests that stress and aging synergistically affect the epigenetic regulation of *FKBP5*, which in turn contributes to inflammation and cardiovascular disease risk (discussed in [57]). Further elucidation of these mechanisms will contribute to our understanding of how psychosocial stressors and the stress response pathways modulate epigenetic mechanisms and impact health outcomes.

This study has many strengths. First, the HRS is a large, nationally representative study with >20 years of longitudinal data and includes a wide range of socio-demographic, lifestyle, and health-related markers. Second, hypothesized relationships were examined using previously validated measures of perceived discrimination, depressive symptoms and biological aging. Nevertheless, study findings need to be interpreted with caution and in light of several limitations. First, the analytic samples used in this study were much smaller than the full HRS sample potentially leading to selection bias. Second, the majority of HRS data were self-reported, potentially leading to non-differential misclassification and measures of association that are biased towards the null value. Third, estimated relationships are prone to confounding bias given the observational nature of the HRS data. Finally, this study involves secondary analysis of existing HRS data and topics consistently covered by the distinct waves of HRS data may or may not have yielded the most relevant predictors of biological aging.

In sum, sustained elevations in depressive symptoms and reasons for perceived discrimination were associated with select measures of epigenetic aging, consistently among women and NHW adults. Furthermore, depressive symptoms acted as potential mediator in the perceived discrimination-epigenetic clock association in most 4-way decomposition models with detected total effect in which perceived discrimination was linked to accelerated epigenetic age.

## Supplementary Materials

Supplementary Methods

Supplementary Figure 1

Supplementary Tables
