# Supplementary material for: Epigenetic clocks and their association with trajectories in perceived discrimination and depressive symptoms among US middle-aged and older adults
Source: Aging (Albany NY). 2022 Jul 1;14(13):5311–44. doi: 10.18632/aging.204150 (PMC9320538; doi:10.18632/aging.204150)
Supplement: Supplementary Methods [file aging-14-204150-s001.pdf]

SUPPLEMENTARY METHODS

Supplementary Method 1: DNA methylation data pre-processing, quality control and the 13 epigenetic clocks:

Data pre-processing and quality control

The *minfi* package in R software was used for data preprocessing, and quality control. 3.4% of the methylation probes (*n* = 29,431 out of 866,091) were removed from the final dataset due to suboptimal performance (using a detection *P*-value threshold of 0.01). Analysis for detection *P*-value failed samples was done after removal of detection *P*-value failed probes. Using a 5% cut-off (*minfi*) we remove 58 samples. We also removed sex mismatched samples and any controls (cell lines, blinded duplicates). High quality methylation data is available for 97.9% samples (*n* = 4,018). Prior to the estimation of the 13 clocks missing beta methylation values were imputed with the mean beta methylation value of the given probe across all samples.

Epigenetic clocks

The thirteen epigenetic clocks were the following:

1. Horvath 1, the first multi-tissue epigenetic clock, was developed using 8,000 samples from 82 Illumina DNA methylation array datasets, incorporating 51 healthy tissues and cell types in order to estimate the DNA methylation age of most tissues and cell types. The clock is defined based on DNA methylation at 353 CpGs that form an aging clock, and shows strong correlation with age (r = 0.96–0.97). Horvath et al. (2013) found DNAm age acceleration was related to multiple types of cancer [1].
2. Hannum: Hannum's epigenetic clock is a blood-based age estimator, based on DNA methylation at 71 CpGs selected from the Illumina 450,000 array (Hannum 2013). Hannum et al. developed this clock based on the whole blood of 656 humans at ages 19 to 101. They reported a strong correlation with age for this clock (r = 0.96) and that the rate of DNAm ageing is influenced by gender and genetic variants [2].
3. Levine: DNAm PhenoAge was developed using composite clinical biomarkers combined into a multi-system measure of biological age, called phenotypic age, which was developed to estimate an individual's mortality risk using 9 markers of tissue and immune function (albumin, creatinine, serum glucose, CRP, lymphocyte percent, mean (red) cell volume, red cell distribution width, alkaline phosphatase, white blood cell count) and age. Phenotypic age was predicted by DNAm PhenoAge based on 513 CpGs in whole blood from the same sample. Levine et al. (2018) found that while this clock was developed using whole blood data, values from all tested tissues and cells are correlated with age and predict mortality better than chronological age-based clocks. DNAm PhenoAge has been shown to predict multiple aging outcomes such as mortality, cancer, healthspan, physical function and Alzheimer's disease; the rate of DNAmPhenoAge acceleration was related to biomarkers such as high CRP, glucose, triglycerides waist-to-hip ratio and low HDL cholesterol [3].
4. Horvath 2: This epigenetic clock, based on 391 CpGs, was developed to better measure the age of human fibroblasts and other skin cells such as keratinocytes, buccal cells, endothelial cells, lymphoblastoid cells, skin, blood, and saliva samples (Horvath et al. 2018). This clock has high age correlations in sorted neurons, glia, brain, liver and bone samples, to predict lifespan and to relate to many age-related conditions. This skin and blood clock shares 45 CpGs with the blood-based clock from Hannum (2013) and 60 CpGs with the pan tissue clock from Horvath (2013). However, epigenetic age acceleration in the skin and blood clock shows only moderate correlations with that of Hannum's and Horvath's 2013 clock [4].
5. Lin: This 99 CpG model was originally derived from the HumanMethylation27K BeadChip data and subsequently modified for the 450,000 BeadChip. It was developed on DNAm profiles of normal blood samples and trained on life expectancy [5–7].
6. Weidner: Weidner et al. (2014) developed a simple DNAm age based on 3 age-related CpGs (cg02228185 in ASPA, cg25809905 in ITGA2B, and cg17861230 in PDE4C), to estimate epigenetic aging in blood. They selected these three CpGs based on recursive feature elimination and pyrosequencing analysis. This clock produced age predictions with average accuracy of 5.4 years [6].
7. Vidal−Bralo: Vidal-Bralo et al. (2016) developed a DNAm age predictor based on 8 CpGs, which were selected as the most informative CpGs in a training set of 390 healthy persons. This clock was developed specifically targeting adults who show slower rates of change compared to pre-adolescents in order to more accurately calibrate DNAm age for adults [8].
8. GrimAge: GrimAge was developed based on the 7 DNAm surrogates of plasma proteins and smoking pack years in a two-stage procedure (Lu et al. 2019). First, they defined surrogate DNAm biomarkers of physiological risk and stress factors with plasma proteins (including adrenomedullin, CRP, plasminogen activation inhibitor 1 (PAI-1) and

growth differentiation factor 15 (GDF15)) and DNAm-based estimator of smoking pack-years. Then, time-to-death was regressed on these biomarkers and an estimator of smoking years to estimate a composite biomarker of lifespan, GrimAge. They named it “DNAm GrimAge” because high values of this measure means grim news in terms of mortality and morbidity risk. Lu et al. (2019) report that the rate of GrimAge-based aging has predictive ability for time to death, coronary heart disease, cancer and age-related conditions [9].

9. Yang et al. (2016) developed a mitotic-like clock using 385 PCGT promoter CpGs. This is based on the DNAm-based age-correlated model called epiTOC (Epigenetic Timer Of Cancer) that features three properties including being constitutively unmethylated across 11 different fetal tissue types, showing age-associated hypermethylation, and targeting the promoters marked by the PRC2 complex in human embryonic stem cells (ESCs). This mitotic-like clock was shown to be universally accelerated in cancer and pre-cancerous lesions [10].
10. Zhang: Zhang et al. (2017) developed a DNAm age based on 10 CpGs that showed a strong association with all-cause mortality, which was selected from replicated results (58 out of 11,063 CpGs with FDR<0.05) from an epigenome-wide association study (EWAS) for all-cause mortality. This epigenetic clock is said to predict disease and mortality better than the original chronological DNAm clocks. This clock specifically identifies those with increased risk of death by cancer and cardiovascular disease [11].
11. Bocklandt: The Bocklandt clock was developed in 2011 using saliva from twin pairs ages 21 to 55 years. The methylation in three sites, EEDARADD, TOM1LI, and NPTX2 genes, was linear with age, and a predictor including two CpGs in the promoter region of EDARADD and NPTX2 explained 73% of the variance in age and predicted age with an average accuracy of 5.2 years [12].
12. Garagnani: Garagnani et al. (2012) used the Illumina Infinium Human Methylation450 BeadChip on whole blood DNA to identify methylation levels of 3 regions, the CpG islands of ELOVL2, FHL2 and PENK genes, strongly correlated with age. This was confirmed using whole blood from 501 persons ages 9 to 99 years and they identified one CpG (cg16867657) in ELOVL2 as a promising biomarker of aging (r = 0.92) [13].
13. A recent blood DNA methylation measure, DunedinPoAm38, was developed to represent individual variation in the pace of biological aging. Based on data from the Illumina 450k Array run on samples from the Dunedin cohort, estimates were derived by using elastic-net regression models to calculate a methylation Pace of Aging (mPoA) score (Belsky et al. 2020). The pace of aging was calculated with composited slopes across the 18 biomarkers that measure the rate of aging in the cardiovascular, metabolic, renal, hepatic, pulmonary, periodontal, and immune systems. Then, the pace of aging composite was scaled to represent the mean trend in the cohort among Dunedin Study members with methylation data at age 38. The Pace of Aging methylation algorithm was trained on 3 waves of biomarker data from participants, including data collected at ages 26, 32, and 38. DunedinPoAm is estimated in years per chronological year (years/chron year) [14].

Supplementary Method 2: CES-D and EOD scales

CES-D scale, 8-items: 2010, 2012 and 2014 waves

C150 During the last 12 months, was there ever a time when you felt sad, blue, or depressed for two weeks or more in a row? 1. YES 3. [VOL] DID NOT FEEL DEPRESSED BECAUSE ON ANTI-DEPRESSANT MEDICATION 5. NO 8. DK 9. RF GO TO C167 BRANCHPOINT

C151 Please think of the two-week period during the last 12 months when these feelings were worst. During that time did the feelings of being sad, blue, or depressed usually last all day long, most of the day, about half the day, or less than half the day? 1. ALL DAY LONG 2. MOST OF THE DAY 3. ABOUT HALF THE DAY 4. LESS THAN HALF THE DAY 8. DK 9. RF GO TO C167 BRANCHPOINT.

C152 During those two weeks, did you feel this way every day, almost every day, or less often than that? 1. EVERY DAY 2. ALMOST EVERY DAY 3. LESS OFTEN 8. DK 9. RF GO TO C167 BRANCHPOINT.

C153 During those two weeks, did you lose interest in most things? [IWER: IF R SAYS USUALLY NO INTEREST IN THINGS: REPEAT Q ADDING: "… more than is usual for you."] 1. YES 5. NO 8. DK 9. RF

C154 Thinking about those same two weeks, did you ever feel more tired out or low in energy than is usual for you? 1. YES 5. NO 8. DK 9. RF

C155 During those same two weeks, did you lose your appetite? 1. YES 5. NO 8. DK 9. RF GO TO C157

C156 Did your appetite increase during those same two weeks? 1. YES 5. NO 8. DK 9. RF C157 Did you have more trouble falling asleep than you usually do during those two weeks? 1. YES 5. NO 8. DK 9. RF GO TO C159

C158 Did that happen every night, nearly every night, or less often during those two weeks? 1. EVERY

NIGHT 2. NEARLY EVERY NIGHT 3. LESS OFTEN 8. DK 9. RF

C159 During that same two-week period, did you have a lot more trouble concentrating than usual? 1. YES 5. NO 8. DK 9. RF

C160 People sometimes feel down on themselves, and no good or worthless. During that two-week period, did you feel this way? 1. YES 5. NO 8. DK 9. RF

C161 Did you think a lot about death ⚜ either your own, someone else's, or death in general ⚜ during those two weeks? 1. YES 5. NO 8. DK 9. RF

Experience of Discrimination, EOD scale: 2010, 2012 and 2014 waves

Q30 – Q31. Perceived Everyday Discrimination (2006, 2008, 2010, 2012; Q29 in 2014 and 2016) This 6-item scale assesses the experience of hassles and chronic stress associated with perceived everyday discrimination. Q31 (Q30 in 2014 and 2016) is a follow-up question which asks about this reason attributed to the experienced discrimination. Similar questions are in MIDUS. The item Q30f was added in 2008 to include a context relevant for older adults.

Source: Williams, D. R., Yu, Y., Jackson, J. S., and Anderson, N. B. (1997). Racial differences in physical and mental health: socio-economic status, stress and discrimination. Journal of Health Psychology, 2, 335-351. 2012: 6 items

(Q30a-Q30f) (In your day-to-day life how often have any of the following things happened to you?)

Q30a You are treated with less courtesy or respect than other people.

Q30b You receive poorer service than other people at restaurants or stores.

Q30c People act as if they think you are not smart.

Q30d People act as if they are afraid of you.

Q30e You are threatened or harassed.

Q30f You receive poorer service or treatment than other people from doctors or hospitals.

Coding: 1 = Almost every day, 2 = At least once a week, 3 = A few times a month, 4 = A few times a year, 5 = Less than once a year, 6 = Never; Scaling:Create an index of discrimination by reverse-coding all items and averaging the scores across all six items. Set the final score to missing if there are more than three items with missing values. Psychometrics: 2014 Alpha = .83, 2012 Alpha = .83, 2010 Alpha = .80, 2008 Alpha = .82

Background:

Sutin, A. R., Stephan, Y., and Terracciano, A. (2016). Perceived discrimination and personality development in adulthood. Developmental Psychology, 52(1), 155–163

Rogers, S. E., Thrasher, A. D., Miao, Y., Boscardin, W. J., and Smith, A. K. (2015). Discrimination in healthcare settings is associated with disability in older adults: health and retirement study, 2008–2012. Journal of General Internal Medicine, 30(10), 1413.

Williams, D.R., Neighbors, H.W., and Jackson, J.S. (2003). Racial/ethnic discrimination and health: Findings from community studies. American Journal of Public Health, 93, 200-208.

EOD scale: Reasons for Perceived discrimination: 2010, 2012 and 2014 waves

Q31. Reasons Attributed for Discrimination (2006, 2008, 2010, 2012; Q30 in2014 and 2016) From 2008 onwards, religion and financial status were added to the attribution categories.

Source: Kessler, R. C., Mickelson, K. D., and Williams, D. R. (1999). The prevalence, distribution, and mental health correlates of perceived discrimination in the United States. Journal of Health and Social Behavior, 40(3), 208-230. 2012: 11 categories (Q31M1 - Q31M11)

(If any of the above (Q30) have happened to you, what do you think were the reasons why these experiences happened to you? (Mark (X) all that apply.)

1 Your ancestry or national origin, 2 Your gender, 3 Your race, 4 Your age, 5 Religion, 6 Your weight, 7 A physical disability, 8 Other aspect of your physical appearance, 9 Your sexual orientation, 10 Your financial status 11 Other Coding: Q31 allows for multiple responses which are delivered in several variables (Q31M1 through Q31M11).

When combined, these variables indicate which attributions and how many attributions were checked. Q31M1 gives the code (1 to 11) for the first attribution a participant checked in the order 1 to 11 as listed above: Q31M2 is the code for the second attribution the participant checked. For example, if the first box a participant checked was age their response on Q31M1 would be coded 4. If this participant also checked financial status, they would have the code 10 for Q31M2. 2008-2016 Coding: 1 = ancestry or national origin, 2 = gender, 3 = race, 4 = age, 5 = religion, 6 = weight, 7 = physical disability, 8 = Other aspect of your physical appearance, 9 = sexual orientation, 10 = financial status, 1 = Other 2006 Coding: 1 = ancestry or national origin, 2 = gender, 3 = race, 4 = age, 5 = weight, 6 = A physical disability, 7 = Other aspect of your physical appearance, 8 = sexual orientation, 9 = Other).

SUPPLEMENTARY REFERENCES

1. Horvath S. DNA methylation age of human tissues and cell types. Genome Biol. 2013; 14:R115. https://doi.org/10.1186/gb-2013-14-10-r115 PMID:24138928

2. Hannum G, Guinney J, Zhao L, Zhang L, Hughes G, Sadda S, Klotzle B, Bibikova M, Fan JB, Gao Y,

Deconde R, Chen M, Rajapakse I, et al. Genome-wide methylation profiles reveal quantitative views of human aging rates. Mol Cell. 2013; 49:359–67. https://doi.org/10.1016/j.molcel.2012.10.016 PMID:23177740

3. Levine ME, Lu AT, Quach A, Chen BH, Assimes TL, Bandinelli S, Hou L, Baccarelli AA, Stewart JD, Li Y, Whitsel EA, Wilson JG, Reiner AP, et al. An epigenetic biomarker of aging for lifespan and healthspan. Aging (Albany NY). 2018; 10:573–91. https://doi.org/10.18632/aging.101414 PMID:29676998

4. Horvath S, Oshima J, Martin GM, Lu AT, Quach A, Cohen H, Felton S, Matsuyama M, Lowe D, Kabacik S, Wilson JG, Reiner AP, Maierhofer A, et al. Epigenetic clock for skin and blood cells applied to Hutchinson Gilford Progeria Syndrome and *ex vivo* studies. Aging (Albany NY). 2018; 10:1758–75. https://doi.org/10.18632/aging.101508 PMID:30048243

5. Lin Q, Wagner W. Epigenetic Aging Signatures Are Coherently Modified in Cancer. PLoS Genet. 2015; 11:e1005334. https://doi.org/10.1371/journal.pgen.1005334 PMID:26110659

6. Weidner CI, Lin Q, Koch CM, Eisele L, Beier F, Ziegler P, Bauerschlag DO, Jöckel KH, Erbel R, Mühleisen TW, Zenke M, Brümmendorf TH, Wagner W. Aging of blood can be tracked by DNA methylation changes at just three CpG sites. Genome Biol. 2014; 15:R24. https://doi.org/10.1186/gb-2014-15-2-r24 PMID:24490752

7. Lin Q, Weidner CI, Costa IG, Marioni RE, Ferreira MR, Deary IJ, Wagner W. DNA methylation levels at individual age-associated CpG sites can be indicative for life expectancy. Aging (Albany NY). 2016; 8:394–401. https://doi.org/10.18632/aging.100908 PMID:26928272

8. Vidal-Bralo L, Lopez-Golan Y, Gonzalez A. Simplified Assay for Epigenetic Age Estimation in Whole Blood of Adults. Front Genet. 2016; 7:126. https://doi.org/10.3389/fgene.2016.00126 PMID:27471517

9. Lu AT, Quach A, Wilson JG, Reiner AP, Aviv A, Raj K, Hou L, Baccarelli AA, Li Y, Stewart JD, Whitsel EA, Assimes TL, Ferrucci L, Horvath S. DNA methylation GrimAge strongly predicts lifespan and healthspan. Aging (Albany NY). 2019; 11:303–27. https://doi.org/10.18632/aging.101684 PMID:30669119

10. Yang Z, Wong A, Kuh D, Paul DS, Rakyan VK, Leslie RD, Zheng SC, Widschwendter M, Beck S, Teschendorff AE. Correlation of an epigenetic mitotic clock with cancer risk. Genome Biol. 2016; 17:205. https://doi.org/10.1186/s13059-016-1064-3 PMID:27716309

11. Zhang Y, Wilson R, Heiss J, Breitling LP, Saum KU, Schöttker B, Holleczek B, Waldenberger M, Peters A, Brenner H. DNA methylation signatures in peripheral blood strongly predict all-cause mortality. Nat Commun. 2017; 8:14617. https://doi.org/10.1038/ncomms14617 PMID:28303888

12. Bocklandt S, Lin W, Sehl ME, Sánchez FJ, Sinsheimer JS, Horvath S, Vilain E. Epigenetic predictor of age. PLoS One. 2011; 6:e14821. https://doi.org/10.1371/journal.pone.0014821 PMID:21731603

13. Garagnani P, Bacalini MG, Pirazzini C, Gori D, Giuliani C, Mari D, Di Blasio AM, Gentilini D, Vitale G, Collino S, Rezzi S, Castellani G, Capri M, et al. Methylation of ELOVL2 gene as a new epigenetic marker of age. Aging Cell. 2012; 11:1132–4. https://doi.org/10.1111/acel.12005 PMID:23061750

14. Belsky DW, Caspi A, Arseneault L, Baccarelli A, Corcoran DL, Gao X, Hannon E, Harrington HL, Rasmussen LJ, Houts R, Huffman K, Kraus WE, Kwon D, et al. Quantification of the pace of biological aging in humans through a blood test, the DunedinPoAm DNA methylation algorithm. Elife. 2020; 9:e54870. https://doi.org/10.7554/eLife.54870 PMID:32367804
