# Supplementary material for: Epigenetic clocks and their association with trajectories in perceived discrimination and depressive symptoms among US middle-aged and older adults
Source: Aging (Albany NY). 2022 Jul 1;14(13):5311–44. doi: 10.18632/aging.204150 (PMC9320538; doi:10.18632/aging.204150)
Supplement: Supplementary Figure 1 [file aging-14-204150-s002.pdf]

SUPPLEMENTARY FIGURE

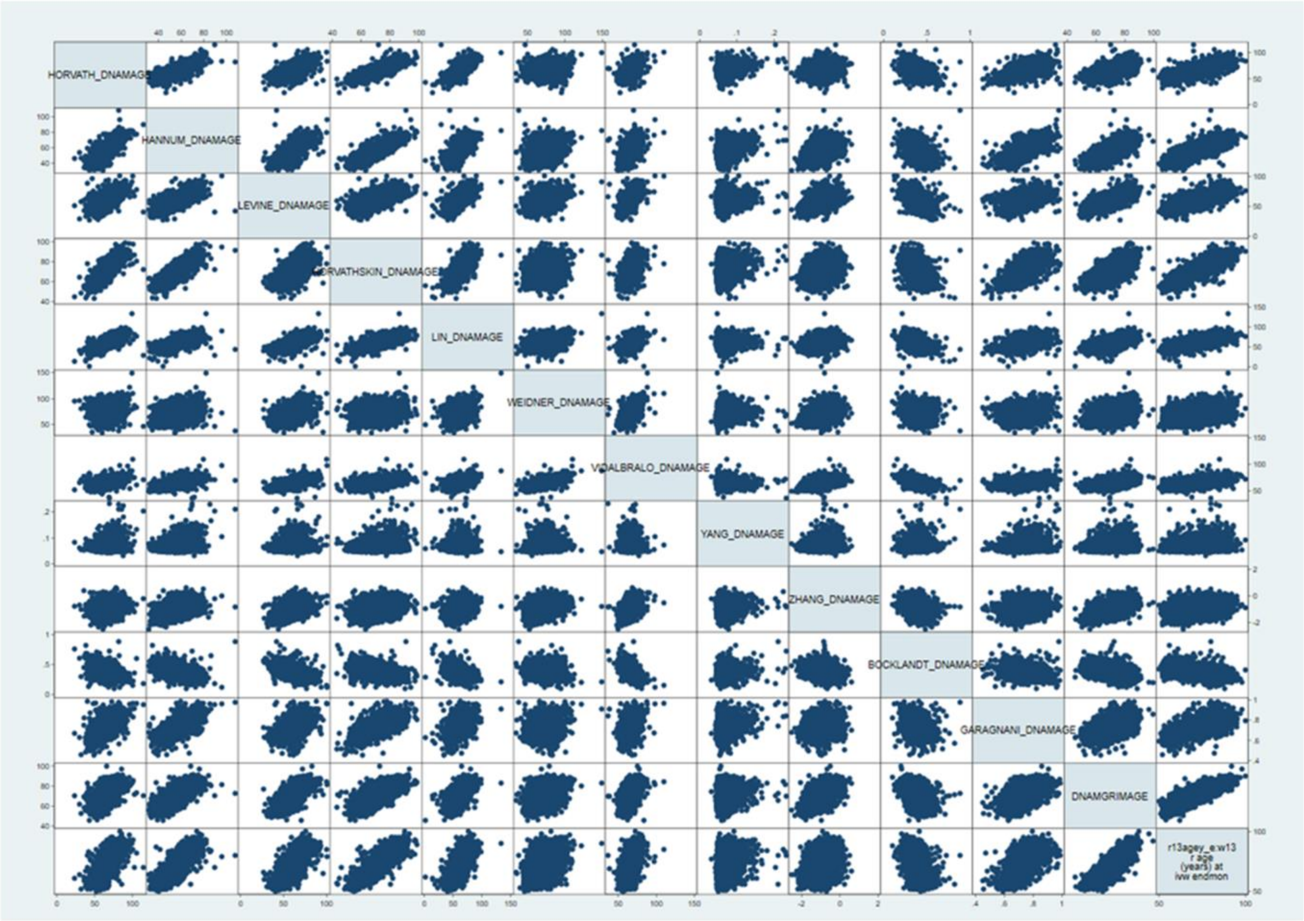

Supplementary Figure 1. **Scatterplot matrix of EPICLOCK measures and age in final selected sample; hrs 2010–2016 (*n* = 2,806).** Note: See list of abbreviations and appendices for detailed description of the epigenetic clock measures.
