# Supplementary material for: Epigenetic clocks and their association with trajectories in perceived discrimination and depressive symptoms among US middle-aged and older adults
Source: Aging (Albany NY). 2022 Jul 1;14(13):5311–44. doi: 10.18632/aging.204150 (PMC9320538; doi:10.18632/aging.204150)
Supplement: Supplementary Tables [file aging-14-204150-s003.pdf]

SUPPLEMENTARY TABLES

Supplementary Table 1. Study sample exposure, mediator and outcomes overall, by sex and by race, HRS 2010-2016[a].

	Overall	Males	Females	NHW	Non-NHW	P^b_{sex}	P^b_{race}
	Mean/% ± SE	Mean/% ± SE	Mean/% ± SE	Mean/% ± SE	Mean/% ± SE		
Trajectory Exposures, 2010-2016[c]							
Trajectory in Experience of Discrimination score							
Stable low	83.0 ± 0.9	80.1 ± 1.4	85.2 ± 1.1	84.1 ± 0.9	77.5 ± 1.9	Ref	Ref
Stable high	17.0 ± 0.9	19.9 ± 1.4	14.7 ± 1.1	15.9 ± 0.9	22.5 ± 1.9	0.008[d]	0.002[d]
Trajectory in Reasons for Experience of Discrimination score							
Stable low	31.3 ± 1.3	31.3 ± 1.8	31.3 ± 1.5	32.7 ± 1.5	24.7 ± 2.1	0.78	0.14
Stable medium	55.5 ± 1.4	56.5 ± 2.0	54.8 ± 1.7	56.1 ± 1.6	52.9 ± 2.7	Ref	Ref
Stable high	13.1 ± 0.7	12.2 ± 1.2	13.9 ± 1.0	11.2 ± 0.8	22.4 ± 1.9	0.31	<0.001[d]
Trajectory in CES-D total score							
Stable low	55.1 ± 1.3	60.7 ± 1.7	50.5 ± 1.7	58.5 ± 1.6	38.4 ± 2.5	Ref	Ref
Stable medium	30.7 ± 1.3	27.4 ± 1.4	33.3 ± 1.7	28.7 ± 1.4	40.2 ± 2.5	<0.001[d]	<0.001[d]
Stable high	14.3 ± 0.7	12.0 ± 0.9	16.2 ± 1.0	12.8 ± 0.8	21.4 ± 2.0	<0.001[d]	<0.001[d]
Point Exposures, 2010−2012							
Experience of discrimination score	9.39 ± 0.12	9.82 ± 0.20	9.05 ± 0.13	9.29 ± 0.12	9.87 ± 0.23	0.002[d]	0.029
Reason for Perceived discrimination score	1.086 ± 0.033	1.090 ± 0.06	1.08 ± 0.04	0.99 ± 0.03	1.56 ± 0.09	0.92	<0.001[d]
CES-D total score	1.23 ± 0.04	1.11 ± 0.06	1.333 ± 0.05	1.12 ± 0.05	1.75 ± 0.11	0.008[d]	<0.001[d]
Point Mediators, 2014−2016							
Experience of discrimination score	9.30 ± 0.10	9.60 ± 0.2	9.06 ± 0.11	9.22 ± 0.11	9.70 ± 0.29	0.008[d]	0.15
Reason for Perceived discrimination score	1.072 ± 0.031	1.026 ± 0.05	1.11 ± 0.04	0.99 ± 0.04	1.49 ± 0.08	0.23	<0.001[d]
CES-D total score	1.23 ± 0.04	1.04 ± 0.057	1.40 ± 0.056	1.15 ± 0.05	1.65 ± 0.10	<0.001[d]	<0.001[d]
Epigenetic Clock Outcomes, 2016							
HORVATH DNAmage	65.7 ± 0.3	66.0 ± 0.4	65.5 ± 0.3	66.1 ± 0.3	63.8 ± 0.4	0.26[d]	<0.001[d]
HANNUM DNAmage	54.4 ± 0.3	55.1 ± 0.4	53.8 ± 0.3	54.9 ± 0.3	52.2 ± 0.4	0.004[d]	<0.001[d]
LEVINE DNAmage	57.0 ± 0.3	57.5 ± 0.4	56.7 ± 0.4	57.3 ± 0.3	55.9 ± 0.5	0.12[d]	0.027
HORVATH 2 DNAmage	69.6 ± 24.9	69.8 ± 0.4	69.4 ± 0.3	69.9 ± 0.3	68.1 ± 0.4	0.41[d]	0.001
LIN DNAmage	58.5 ± 0.3	58.9 ± 0.5	58.2 ± 0.3	59.0 ± 0.4	56.2 ± 0.5	0.18[d]	<0.001[d]
WEIDNER DNAmage	67.0 ± 0.3	67.4 ± 0.5	66.7 ± 0.4	67.2 ± 0.4	66.2 ± 0.5	0.27	*0.095*
VIDAL-BRALO DNAmage	63.8 ± 0.2	64.6 ± 0.2	63.2 ± 0.2	64.2 ± 0.2	62.1 ± 0.3	<0.001[d]	<0.001[d]
YANG DNAmage	0.067 ± 0.0003	0.067 ± 0.001	0.068 ± 0.000	0.066 ± 0.000	0.074 ± 0.001	0.44	<0.001[d]
ZHANG DNAmage	−1.105 ± 0.013	−0.989 ± 0.02	−1.197 ± 0.015	−1.085 ± 0.015	−1.202 ± 0.025	<0.001[d]	<0.001[d]
BOCKLANDT DNAmage	0.388 ± 0.002	0.380 ± 0.003	0.395 ± 0.003	0.382 ± 0.002	0.419 ± 0.004	<0.001[d]	<0.001[d]
GARAGNANI DNAmage	0.714 ± 0.002	0.708 ± 0.003	0.719 ± 0.002	0.716 ± 0.002	0.705 ± 0.003	0.001[d]	0.020
DNAm GRIMAGE	67.4 ± 0.2	68.8 ± 0.3	66.2 ± 0.3	67.5 ± 0.3	66.8 ± 0.43	<0.001[d]	0.18[d]
MPOA	1.072 ± 0.002	1.072 ± 0.003	1.059 ± 0.003	1.061 ± 0.002	1.080 ± 0.006	0.001[d]	0.004[d]

Abbreviations: CES-D: Centers for Epidemiological Studies-Depression; DNAm: DNA methylation; DNAmage DNA methylation age; GBTM Group-based trajectory models. [a]Values are means ± SE or % ± SE, overall and across sex or race/ethnicity groups for main baseline and follow-up exposures and outcomes (See Methods section and OSM for details), taking into account sampling weights and sampling design complexity in multiple imputed data. Italicized findings have $p < 0.10$ but >0.05. [b]Based on linear or multinomial logit models with sex or race as the only predictors of continuous and categorical variables, respectively, taking into account sampling weights and sampling design complexity in multiple imputed data. [c]Trajectory exposures determined using GBTM (See Methods section for detail). [d]P < 0.05 after further adjustment of other demographic variables, including age in 2016, birth cohort, sex and race.

Supplementary Table 2. Trajectories in experience of discrimination, reasons for perceived discrimination and depressive symptoms (2010-2016) and their association with 13 epigenetic clocks (2016): Multiple OLS linear regression models, stratified analysis: HRS 2010-2016[a,b].

Y = Epigenetic clock	X = Experience of discrimination score trajectory		X = Reasons for perceived discrimination trajectory				X = CES-D total score trajectory			
	High vs. Low		Medium vs. Low		High vs. Low		Medium vs. Low		High vs. Low	
	β ± SE	*P*	β ± SE	*P*	β ± SE	*P*	β ± SE	*P*	β ± SE	*P*
HANNUM DNAmage										
Model 1										
NHW	+0.090 ± 0.306	0.77	__		__		+0.007 ± 0.258	0.98	*+0.674 ± 0.375*	*0.078*
Non-White	−0.553 ± 0.490	0.26	__		__		0.758 ± 0.618	0.23	1.166 ± 0.577	0.048
Model 2										
NHW	−0.0165 ± 0.295	0.96	__		__		−0.0601 ± 0.252	0.81	+0.473 ± 0.385	0.22
Non-White	−0.700 ± 0.470	0.14	__		__		+0.722 ± 0.646	0.27	*+1.073 ± 0.555*	*0.060*
Model 3										
NHW	−0.225 ± 0.316	0.48	__		__		−0.164 ± 0.273	0.55	+0.224 ± 0.394	0.57
Non-White	−0.795 ± 0.478	0.10	__		__		+0.517 ± 0.638	0.42	0.631 ± 0.714	0.38
HORVATH 2 DNAmage										
Model 1										
NHW	+0.492 ± 0.322	0.13	__		__		__		__	
Non-White	−0.002 ± 0.545	0.99	__		__		__		__	
Model 2										
NHW	+0.359 ± 0.316	0.26	__		__		__		__	
Non-White	+0.066 ± 0.545	0.91	__		__		__		__	
Model 3										
NHW	+0.170 ± 0.337	0.62	__		__		__		__	
Non-White	−0.190 ± 0.504	0.71	__		__		__		__	
LIN DNAmage										
Model 1										
Males	−0.958 ± 0.457	0.041	−0.455 ± 0.619	0.47	*−1.207 ± 0 .664*	*0.075*	+0.462 ± 0.490	0.35	−0.829 ± 0.670	0.22
Females	*+1.397 ± 0.736*	*0.063*	+0.441 ± 0.578	0.45	0.682 ± 0.790	0.39	+1.849 ± 0.483	<0.001[c]	+1.506 ± 0.559	0.009[c]
Model 2										
Males	*−0.823 ± 0.446*	*0.070*	−0.452 ± 0.600	0.45	−1.111 ± 0.696	0.12	+0.656 ± 0.500	0.20	−0.555 ± 0.623	0.38
Females	*+1.395 ± 0.726*	*0.060*	+0.405 ± 0.574	0.48	+0.640 ± 0.793	0.42	+1.889 ± 0.470	<0.001	1.618 ± 0.590	0.008
Model 3										
Males	−0.867 ± 0.412	0.040	−0.593 ± 0.576	0.31	−1.574 ± 0.691	0.027	+0.586 ± 0.490	0.24	−0.711 ± 0.722	0.33
Females	+1.203 ± 0.749	0.11	+0.392 ± 0.574	0.50	+0.291 ± 0.757	0.70	+1.837 ± 0.489	<0.001	1.314 ± 0.636	0.044
WEIDNER DNAmage										
Model 1										
NHW	+0.153 ± 0.743	0.84	−0.534 ± 0.588	0.37	−0.913 ± 0.901	0.32	__		__	
Non-White	−1.086 ± 1.011	0.29	−1.841 ± 1.445	0.21	−1.429 ± 1.596	0.38	__		__	
Model 2										
NHW	+0.198 ± 0.769	0.80	−0.486 ± 0.599	0.42	−0.911 ± 0.887	0.31	__		__	
Non-White	−0.939 ± 1.012	0.36	−2.000 ± 1.453	0.18	−1.494 ± 1.593	0.35	__		__	
Model 3										
NHW	+0.297 ± 0.760	0.70	−0.549 ± 0.600	0.36	−1.066 ± 0.931	0.26	__		__	
Non-White	−0.747 ± 1.057	0.48	−1.953 ± 1.315	0.14	−1.668 ± 1.536	0.28	__		__	
VIDAL-BRALO DNAmage										

Model 1								
Males	*−0.874 ± 0.498*	*0.085*	—	—	—		—	
Females	+1.059 ± 0.420	0.015[c]	—	—	—		—	
NHW	+0.065 ± 0.391	0.87	—	—	—		—	
Non-White	+0.150 ± 0.585	0.80	—	—	—		—	
Model 2								
Males	−0.792 ± 0.492	0.11	—	—	—		—	
Females	+0.914 ± 0.420	0.034	—	—	—		—	
NHW	−0.007 ± 0.393	0.99	—	—	—		—	
Non-White	+0.203 ± 0.553	0.71	—	—	—		—	
Model 3								
Males	*−0.803 ± 0.457*	*0.084*	—	—	—		—	
Females	+0.697 ± 0.443	0.12	—	—	—		—	
NHW	−0.165 ± 0.375	0.66	—	—	—		—	
Non-White	+0.118 ± 0.481	0.81	—	—	—		—	
YANG DNAmage								
Model 1								
NHW	−0.001 ± 0.001	0.26	—	—	—		—	
Non-White	+0.003 ± 0.002	0.11	—	—	—		—	
Model 2								
NHW	−0.0012 ± 0.0009	0.16	—	—	—		—	
Non-White	+0.00228 ± 0.0019	0.24	—	—	—		—	
Model 3								
NHW	*−0.0015 ± 0.0009*	*0.085*	—	—	—		—	
Non-White	+0.0016 ± 0.0019	0.41	—	—	—		—	
ZHANG DNAmage								
Model 1								
NHW	+0.041 ± 0.036	0.26	—	—	—		—	
Non-White	−0.064 ± 0.058	0.27	—	—	—		—	
Model 2								
NHW	+0.019 ± 0.035	0.59	—	—	—		—	
Non-White	+0.002 ± 0.002	0.24	—	—	—		—	
Model 3								
NHW	−0.0004 ± 0.0301	0.99	—	—	—		—	
Non-White	−0.120 ± 0.058	0.043	—	—	—		—	
DNAm GRIMAGE								
Model 1								
NHW	—		—	—	+0.586 ± 0.282	0.043	1.885 ± 0.408	<0.001
Non-White	—		—	—	+1.464 ± 0.574	0.014[c]	1.934 ± 0.582	0.002[c]
Model 2								
NHW	—		—	—	*+0.417 ± 0.248*	*0.098*	1.241 ± 0.406	0.004
Non-White	—		—	—	+0.958 ± 0.590	0.11	1.392 ± 0.584	0.021
Model 3								
NHW	—		—	—	−0.027 ± 0.212	0.90	+0.579 ± 0.354	0.11
Non-White	—		—	—	+0.253 ± 0.408	0.54	+0.426 ± 0.501	0.40
MPOA								
Model 1								
NHW	—		—	—	+0.586 ± 0.282	0.043	+1.885 ± 0.408	<0.001

Non-White	__	__	__	*+0.017 ± 0.0100*	*0.089*	*+0.024 ± 0.012*	*0.055*
Model 2							
NHW	__	__	__	+0.0038 ± 0.0056	0.50	0.0119 ± 0.0083	0.16
Non-White	__	__	__	+0.0077 ± 0.0112	0.50	+0.0109 ± 0.0127	0.40
Model 3							
NHW	__	__	__	−0.0021 ± 0.0051	0.68	+0.0032 ± 0.0081	0.70
Non-White	__	__	__	−0.0012 ± 0.0104	0.91	−0.0023 ± 0.0118	0.85

Abbreviations: CES−D: Centers for Epidemiological Studies−Depression; DNAm: DNA methylation; DNAmage: DNA methylation age; GBTM: Group−based trajectory models; HRS Health and Retirement Study; NHW: Non−Hispanic White. [a]OLS regression models with epigenetic clocks as alternative outcomes and trajectories in EOD, RPD and CES−D scores as alternative exposures. Subpopulation sample size N = 2,728, accounting for sampling weights, PSU and strata. Aside from fixed covariates and age which is measured in 2016, all other covariates were measured in 2010. Stratified analysis by sex and/or race was presented only when $p < 0.05$ for Exposure*sex or Exposure*race for at least one contrast in the unstratified model with 2−way interaction terms. [b]Model 1 adjusted for sex, age in 2016, birth cohort and race/ethnicity; Model 2 further adjusted Model 1 for education and total wealth in 2010; Model 3 further adjusted Model 2 for the remaining socio−demographic, lifestyle and health−related factors (See Covariates section for detail). Italicized findings have $p < 0.10$ but >0.05. [c]Passed correction for multiple testing at type I error of 0.05 (corrected p−value accounting for exposure type: 0.017), applied only to Model 1.

Supplementary Table 3. Experience of discrimination (EOD: 2010/2012) → depressive symptoms (CES−D: 2014/2016) → epigenetic clocks (2016): 4−way mediation analysis, overall and by sex and race, reduced model: HRS 2010–2016.

Y = Epigenetic clock	Overall		Males		Females		NHW		Non-Whites	
	β ± SE	P	β ± SE	P	β ± SE	P	β ± SE	P	β ± SE	P
HORVATH DNAmage										
TE	−0.0007268 ± 0.0348995	0.98	−0.0087547 ± 0.052328	0.87	−0.0000364 ± 0.0477565	0.99	−0.0009221 ± 0.0425617	0.98	0.0043068 ± 0.0600669	0.94
CDE	0.0004887 ± 0.0395531	0.99	0.0053674 ± 0.056734	0.93	−0.0071211 ± 0.0553038	0.90	0.0011853 ± 0.0471137	0.98	0.0056666 ± 0.0729816	0.94
INTREF	−0.00184 ± 0.0114849	0.87	0.002116 ± 0.0137432	0.88	−0.0018813 ± 0.0173885	0.91	−0.0024273 ± 0.0121818	0.84	−0.0030151 ± 0.0278226	0.91
INTMED	−0.0002063 ± 0.0012877	0.87	0.0002752 ± 0.0017872	0.88	−0.0002035 ± 0.0018806	0.91	−0.0003141 ± 0.0015762	0.84	−0.0002463 ± 0.0022712	0.91
PIE	0.0008308 ± 0.0093485	0.93	−0.0165133 ± 0.015121	0.28	0.0091695 ± 0.0126106	0.47	0.000634 ± 0.0111854	0.96	0.0019016 ± 0.0171206	0.91
HANNUM DNAmage										
TE	−0.0089446 ± 0.0280294	0.75	−0.0107057 ± 0.0424706	0.80	−0.0070387 ± 0.0381099	0.85	0.0221764 ± 0.0344324	0.52	−0.0704069 ± 0.0468303	0.13
CDE	−0.0297586 ± 0.031751	0.35	−0.0238521 ± 0.0460677	0.61	−0.0339888 ± 0.0441126	0.44	0.0060065 ± 0.0381	0.88	*−0.1056368 ± 0.0568774*	*0.063*
INTREF	0.0072604 ± 0.0092252	0.43	0.0035162 ± 0.0111624	0.75	0.0107238 ± 0.0138821	0.44	0.0034773 ± 0.0098544	0.72	0.0204714 ± 0.0217358	0.35
INTMED	0.000814 ± 0.0010355	0.43	0.0004572 ± 0.0014519	0.75	0.0011598 ± 0.0015048	0.44	0.0004499 ± 0.0012753	0.72	0.001671 ± 0.001787	0.35
PIE	*0.0127396 ± 0.0075701*	*0.092*	0.009173 ± 0.0122279	0.45	0.0150665 ± 0.0101503	0.14	0.0122427 ± 0.0091175	0.18	0.0130875 ± 0.0134723	0.33
LEVINE DNAmage										
TE	0.0396396 ± 0.0371932	0.29	0.0455454 ± 0.0549787	0.41	0.0451332 ± 0.0513226	0.38	*0.0864305 ± 0.0454536*	*0.057*	−0.0571024 ± 0.0637075	0.37
CDE	0.0224769 ± 0.0418809	0.59	0.0329356 ± 0.059269	0.58	0.0229713 ± 0.0590678	0.70	0.0731105 ± 0.0497503	0.14	−0.0996989 ± 0.0773821	0.20
INTREF	−0.0259646 ± 0.012219	0.034	*−0.0271444 ± 0.0145359*	*0.062*	−0.0247268 ± 0.0186215	0.18	−0.040623 ± 0.0130955	0.002	0.0239875 ± 0.0295548	0.42
INTMED	−0.0029109 ± 0.0013822	0.035	*−0.0035296 ± 0.0019092*	*0.064*	−0.0026742 ± 0.0020274	0.19	−0.0052556 ± 0.0017347	0.002	0.001958 ± 0.0024253	0.42
PIE	0.0460382 ± 0.0105328	<0.001	0.0432839 ± 0.0164261	0.008	0.0495628 ± 0.0143735	0.001	0.0591986 ± 0.0130336	0.000	0.0166511 ± 0.0183062	0.36
HORVATH 2 DNAmage										
TE	0.0278322 ± 0.0238071	0.24	0.0344477 ± 0.0367005	0.35	0.0280118 ± 0.0319923	0.38	0.068629 ± 0.0291545	0.019	−0.0594043 ± 0.0403658	0.14
CDE	0.0197312 ± 0.0269738	0.46	0.0280313 ± 0.0397602	0.48	0.0163424 ± 0.0370533	0.66	0.0646395 ± 0.0322449	0.045	*−0.0867864 ± 0.0489916*	*0.076*
INTREF	0.0005217 ± 0.0078321	0.95	−0.007233 ± 0.0096498	0.45	0.0068647 ± 0.0116561	0.56	−0.0065217 ± 0.0083473	0.44	0.0283076 ± 0.018786	0.13
INTMED	0.0000585 ± 0.0008781	0.95	−0.0009405 ± 0.0012568	0.45	0.0007424 ± 0.0012623	0.56	−0.0008437 ± 0.0010816	0.44	0.0023106 ± 0.0015616	0.13
PIE	0.0075208 ± 0.0064022	0.24	0.0145898 ± 0.0106449	0.171	0.0040624 ± 0.0084356	0.63	0.011355 ± 0.0077284	0.142	−0.0032362 ± 0.0114978	0.78
LIN DNAmage										
TE	0.0109669 ± 0.0408529	0.79	−0.0453771 ± 0.0602042	0.45	0.0611492 ± 0.0564145	0.28	0.0502662 ± 0.0504288	0.32	−0.0612604 ± 0.0675806	0.37
CDE	−0.0049886 ± 0.0462897	0.91	−0.0397808 ± 0.0652912	0.54	0.0348879 ± 0.065273	0.59	0.04573 ± 0.0557751	0.41	−0.1161494 ± 0.0820279	0.16
INTREF	0.0034203 ± 0.0134416	0.80	0.0064654 ± 0.0158241	0.68	0.0005928 ± 0.0205226	0.98	−0.0126346 ± 0.0144425	0.38	*0.0519658 ± 0.0314905*	*0.099*
INTMED	0.0003835 ± 0.0015071	0.80	0.0008407 ± 0.0020586	0.68	0.0000641 ± 0.0022195	0.98	−0.0016346 ± 0.0018721	0.38	0.0042416 ± 0.0026266	0.11

PIE	0.0121517 ± 0.0109815	0.268	−0.0129023 ± 0.0173296	0.457	*0.0256044 ± 0.0150755*	*0.089*	0.0188054 ± 0.0133578	0.159	−0.0013184 ± 0.019236	0.945
WEIDNER DNAmage										
TE	−0.0744727 ± 0.0578073	0.20	−0.0411406 ± 0.0859564	0.63	−0.0719682 ± 0.0795334	0.37	−0.0604088 ± 0.0711575	0.40	−0.0814831 ± 0.0966803	0.40
CDE	*−0.1150437 ± 0.0655048*	*0.079*	−0.0603463 ± 0.0930547	0.52	−0.1361453 ± 0.0920582	0.14	−0.0804088 ± 0.078736	0.31	−0.1832858 ± 0.1172614	0.12
INTREF	0.023837 ± 0.0190515	0.21	−0.0201874 ± 0.0226033	0.37	0.0591688 ± 0.0291271	0.042	−0.0017923 ± 0.0203577	0.93	0.0927537 ± 0.0451729	0.040
INTMED	0.0026724 ± 0.0021426	0.21	−0.002625 ± 0.002946	0.37	0.0063991 ± 0.0031993	0.045	−0.0002318 ± 0.0026339	0.93	0.0075711 ± 0.0038125	0.047
PIE	0.0140617 ± 0.0155209	0.37	*0.0420182 ± 0.0250677*	*0.094*	−0.0013908 ± 0.0209327	0.95	0.0220242 ± 0.0188047	0.24	0.001478 ± 0.0275031	0.96
VIDAL−BRALO DNAmage										
TE	−0.0032406 ± 0.0271298	0.91	−0.0110142 ± 0.0406386	0.79	0.0178137 ± 0.0371223	0.63	0.012165 ± 0.0334859	0.72	−0.0303985 ± 0.0450071	0.50
CDE	−0.0191508 ± 0.0307184	0.53	−0.0139461 ± 0.0438741	0.75	−0.013233 ± 0.0429826	0.76	0.0053836 ± 0.0369986	0.88	−0.0765071 ± 0.0546499	0.16
INTREF	0.0006006 ± 0.0089194	0.95	*−0.0208067 ± 0.0107698*	*0.053*	0.0196147 ± 0.0135573	0.15	−0.0106238 ± 0.0095887	0.27	0.0344447 ± 0.0209791	0.10
INTMED	0.0000673 ± 0.001	0.95	*−0.0027055 ± 0.0014155*	*0.056*	0.0021213 ± 0.0014779	0.15	−0.0013745 ± 0.0012444	0.27	0.0028115 ± 0.0017496	0.11
PIE	0.0152423 ± 0.0073573	0.038	0.0264441 ± 0.011985	0.027	0.0093106 ± 0.0098205	0.34	0.0187797 ± 0.0089568	0.036	0.0088523 ± 0.0128794	0.49
YANG DNAmage										
TE	−0.0000457 ± 0.0000981	0.64	0.0000648 ± 0.0001603	0.69	−0.0001136 ± 0.0001255	0.37	*−0.000202 ± 0.0001191*	*0.090*	*0.0003161 ± 0.0001701*	*0.063*
CDE	−0.0001041 ± 0.0001112	0.35	0.0000274 ± 0.0001737	0.87	−0.0002024 ± 0.0001454	0.16	*−0.000250 ± 0.000132*	*0.058*	0.0002485 ± 0.0002066	0.23
INTREF	0.000026 ± 0.0000323	0.42	−0.0000233 ± 0.0000421	0.58	0.0000713 ± 0.0000459	0.12	0.0000149 ± 0.0000341	0.66	0.0000242 ± 0.0000788	0.76
INTMED	0.0000029 ± 0.0000036	0.42	−0.000003 ± 0.0000055	0.58	0.0000077 ± 0.000005	0.12	0.0000019 ± 0.0000044	0.66	0.000002 ± 0.0000064	0.76
PIE	0.0000295 ± 0.0000264	0.26	0.0000638 ± 0.0000465	0.17	0.0000098 ± 0.0000331	0.77	0.0000306 ± 0.0000314	0.33	0.0000414 ± 0.0000488	0.40
ZHANG DNAmage										
TE	0.0006654 ± 0.0022779	0.77	0.0022686 ± 0.0034092	0.51	−0.0006544 ± 0.0031191	0.83	0.0011064 ± 0.0027764	0.69	−0.0003756 ± 0.003924	0.92
CDE	−0.0016618 ± 0.0025737	0.52	0.0001181 ± 0.0036842	0.97	−0.0034306 ± 0.0036031	0.34	−0.0014068 ± 0.003064	0.65	−0.002299 ± 0.0047551	0.63
INTREF	0.0000285 ± 0.0007473	0.97	−0.0003537 ± 0.0008929	0.69	0.0004281 ± 0.0011331	0.71	0.000111 ± 0.0007923	0.89	−0.0000962 ± 0.0018132	0.96
INTMED	0.0000032 ± 0.0000838	0.97	−0.000046 ± 0.0001162	0.69	0.0000463 ± 0.0001226	0.71	0.0000144 ± 0.0001025	0.89	−0.0000079 ± 0.000148	0.96
PIE	0.0022954 ± 0.0006342	<0.001	0.0025503 ± 0.0010164	0.012	0.0023018 ± 0.0008531	0.007	0.0023878 ± 0.0007607	0.002	*0.0020274 ± 0.0011535*	*0.079*
BOCKLANDT DNAmage										
TE	0.0005824 ± 0.0003788	0.12	0.0008922 ± 0.0005731	0.12	0.0002264 ± 0.0005152	0.66	0.0001703 ± 0.0004443	0.70	+0.0015051 ± 0.0007172	0.036
CDE	0.000607 ± 0.0004292	0.16	0.0009645 ± 0.000621	0.12	0.0002283 ± 0.0005966	0.70	0.0001759 ± 0.0004914	0.72	*+0.0016907 ± 0.0008714*	*0.052*
INTREF	0.000073 ± 0.0001247	0.56	0.0001149 ± 0.0001507	0.45	0.000063 ± 0.0001876	0.74	0.0001355 ± 0.0001273	0.29	−0.0002042 ± 0.0003326	0.54
INTMED	0.0000082 ± 0.000014	0.56	0.0000149 ± 0.0000196	0.45	0.0000068 ± 0.0000203	0.74	0.0000175 ± 0.0000165	0.29	−0.0000167 ± 0.0000272	0.54
PIE	−0.0001058 ± 0.0001018	0.30	−0.0002021 ± 0.0001658	0.22	−0.0000717 ± 0.0001359	0.60	−0.0001587 ± 0.0001176	0.18	0.0000353 ± 0.0002044	0.86
GARAGNANI DNAmage										
TE	0.0002897 ± 0.0002931	0.32	0.000342 ± 0.0004375	0.43	0.0003304 ± 0.000402	0.41	0.0001916 ± 0.0003633	0.60	0.0006222 ± 0.0004769	0.19
CDE	0.0001534 ± 0.0003321	0.64	0.0002808 ± 0.0004744	0.55	0.0001287 ± 0.0004656	0.78	0.0001363 ± 0.0004022	0.74	0.00034 ± 0.0005787	0.56
INTREF	0.0000505 ± 0.0000964	0.60	−0.0000515 ± 0.000115	0.65	0.0001287 ± 0.0001465	0.38	0.0000102 ± 0.000104	0.92	0.000084 ± 0.0002207	0.70
INTMED	0.0000057 ± 0.0000108	0.60	−0.0000067 ± 0.000015	0.65	0.0000139 ± 0.0000159	0.38	0.0000013 ± 0.0000135	0.92	0.0000069 ± 0.000018	0.70
PIE	0.00008 ± 0.0000787	0.31	0.0001194 ± 0.0001262	0.34	0.0000591 ± 0.000106	0.58	0.0000438 ± 0.0000956	0.65	0.0001913 ± 0.0001385	0.17
DNAm GRIMAGE										
TE	0.01887 ± 0.0236422	0.42	−0.0159779 ± 0.0362651	0.66	0.0512312 ± 0.0318333	0.11	0.0168434 ± 0.0283208	0.55	0.0243815 ± 0.0425455	0.57
CDE	−0.0138229 ± 0.0266884	0.61	−0.0515983 ± 0.0392338	0.19	0.0226063 ± 0.0366671	0.54	−0.0179571 ± 0.0312126	0.57	−0.0031184 ± 0.0515369	0.95
INTREF	0.0042824 ± 0.0077517	0.58	0.0081435 ± 0.0095277	0.39	−0.0028459 ± 0.0115296	0.81	0.0044353 ± 0.0080756	0.58	0.0031257 ± 0.0196526	0.87
INTMED	0.0004801 ± 0.0008696	0.58	0.0010589 ± 0.0012415	0.39	−0.0003078 ± 0.0012472	0.81	0.0005738 ± 0.0010456	0.58	0.0002551 ± 0.0016044	0.87
PIE	0.0279303 ± 0.0066752	<0.001	0.026418 ± 0.0108004	0.014	0.0317785 ± 0.0089605	<0.001	0.0297914 ± 0.0079126	<0.001	*0.0241191 ± 0.0125865*	*0.055*
MPOA										
TE	0.0005906 ± 0.0004832	0.22	0.0003824 ± 0.0007391	0.61	0.0007075 ± 0.0006528	0.28	0.0005948 ± 0.0005848	0.31	0.0006003 ± 0.0008484	0.48
CDE	0.0003396 ± 0.000546	0.53	0.0000211 ± 0.0008011	0.98	0.0005741 ± 0.0007523	0.45	0.0003818 ± 0.0006451	0.55	0.0002085 ± 0.0010286	0.84
INTREF	−0.0001603 ± 0.0001587	0.31	0.0000525 ± 0.0001941	0.79	−0.0003636 ± 0.0002374	0.13	−0.0002154 ± 0.0001673	0.20	−0.000001 ± 0.0003922	1.00
INTMED	−0.000018 ± 0.0000178	0.31	0.0000068 ± 0.0000252	0.79	−0.0000393 ± 0.0000259	0.13	−0.0000279 ± 0.0000217	0.20	−0.0000001 ± 0.000032	1.00

PIE	0.0004293 ± 0.0001333	0.001	0.0003019 ± 0.0002146	0.16	0.0005363 ± 0.0001798	0.003	0.0004563 ± 0.000159	0.004	0.0003928 ± 0.0002479	0.11

Abbreviations: CDE: Controlled Direct Effect; CES–D: Centers for Epidemiological Studies–Depression; DNAm: DNA methylation; DNAmage: DNA methylation age; INTMED: Mediated Interaction; INTREF: Interaction referent; PIE: Pure Indirect Effect; TE: Total Effect. [a]OLS regression models with epigenetic clocks as alternative outcomes and point PD measured in 2010–2012 as exposures and CES–D scores measured in 2014–2016 as a potential mediator, sample size N = 2,806, four–way mediation analysis. Stratified analysis by sex and/or race was also presented. Italicized findings have p < 0.10 but >0.05. [b]Exogenous variables are the ones included in Table 2, Model 1, as covariates. See Covariates section for detail.

Supplementary Table 4. Reasons for Perceived discrimination (RPD: 2010/2012) → depressive symptoms (CES–D: 2014/2016) → epigenetic clocks (2016): 4–way mediation analysis, overall and by sex and race, reduced model: HRS 2010–2016.

Y = Epigenetic clock	Overall		Males		Females		NHW		Non-Whites	
	β ± SE	*P*	β ± SE	*P*	β ± SE	*P*	β ± SE	*P*	β ± SE	*P*
HORVATH DNAmage										
TE	−0.0535582 ± 0.0903702	0.55	−0.1443057 ± 0.1376973	0.30	0.0134141 ± 0.119973	0.91	0.0086883 ± 0.1231711	0.94	−0.1247175 ± 0.1297758	0.34
CDE	−0.0854419 ± 0.1074138	0.43	−0.1514943 ± 0.1622281	0.35	−0.0300265 ± 0.1444936	0.84	−0.000204 ± 0.1432616	1.00	−0.2100947 ± 0.1641615	0.20
INTREF	0.0381976 ± 0.0369128	0.30	0.0427118 ± 0.0587631	0.47	0.0309235 ± 0.049463	0.53	0.0171541 ± 0.0421285	0.68	0.09293 ± 0.0794797	0.24
INTMED	0.009544 ± 0.0092575	0.30	0.0104492 ± 0.0144552	0.47	0.0078196 ± 0.0125332	0.53	0.0066735 ± 0.0163971	0.68	0.010264 ± 0.0091021	0.26
PIE	−0.015858 ± 0.0226344	0.48	−0.0459724 ± 0.0347272	0.19	0.0046975 ± 0.030629	0.88	−0.0149353 ± 0.0357075	0.68	−0.0178168 ± 0.0262914	0.50
HANNUM DNAmage										
TE	0.0344417 ± 0.0727078	0.67	0.0324369 ± 0.1118175	0.77	0.0363654 ± 0.0959864	0.71	*0.1745326 ± 0.099781*	*0.080*	−0.1477397 ± 0.1013168	0.15
CDE	0.021214 ± 0.0862353	0.81	0.0304539 ± 0.1317737	0.82	0.0167592 ± 0.1152535	0.88	0.1698007 ± 0.1158047	0.14	−0.1832859 ± 0.1280906	0.15
INTREF	−0.0305501 ± 0.029635	0.30	−0.0263818 ± 0.0477045	0.58	−0.0344673 ± 0.0394759	0.38	−0.0464508 ± 0.0341564	0.17	0.0067812 ± 0.0618586	0.91
INTMED	−0.0076332 ± 0.0074321	0.30	−0.0064541 ± 0.0117079	0.58	−0.0087157 ± 0.0100219	0.38	−0.0180713 ± 0.0133558	0.18	0.0007492 ± 0.0068352	0.91
PIE	0.0514111 ± 0.018768	0.006	0.034819 ± 0.0281238	0.22	0.0627892 ± 0.0254858	0.014	0.069254 ± 0.0295713	0.019	0.0280158 ± 0.0213552	0.19
LEVINE DNAmage										
TE	0.0988918 ± 0.0962204	0.30	0.0254435 ± 0.144451	0.86	0.1620573 ± 0.1289253	0.21	0.3448994 ± 0.1310714	0.009	−0.1949576 ± 0.1375862	0.16
CDE	0.0762701 ± 0.1138207	0.50	0.0371221 ± 0.1697287	0.83	0.1329307 ± 0.1543623	0.39	0.3298103 ± 0.1514449	0.029	−0.2608407 ± 0.1740669	0.13
INTREF	−0.0604681 ± 0.0391635	0.12	−0.0719209 ± 0.0616121	0.24	−0.06932 ± 0.0529462	0.19	−0.1010561 ± 0.044912	0.024	0.0302025 ± 0.0840824	0.72
INTMED	−0.0151085 ± 0.0098667	0.13	−0.017595 ± 0.0152863	0.25	−0.0175289 ± 0.0135079	0.19	−0.039315 ± 0.0177146	0.026	0.0033365 ± 0.0093209	0.72
PIE	0.0981983 ± 0.0256588	0.000	0.0778372 ± 0.0376508	0.039	0.1159755 ± 0.0353643	0.001	0.1554602 ± 0.0404599	<0.001	0.0323441 ± 0.0286036	0.258
HORVATH 2 DNAmage										
TE	*0.1026264 ± 0.0616615*	*0.096*	*0.1783227 ± 0.0963915*	*0.064*	0.0447054 ± 0.0804603	0.59	0.2815875 ± 0.0842897	0.001	−0.1212641 ± 0.0871261	0.16
CDE	0.0961187 ± 0.0732552	0.19	0.1803375 ± 0.1136303	0.11	0.0279376 ± 0.0968616	0.77	0.2899032 ± 0.0979432	0.003	−0.1694663 ± 0.1103595	0.13
INTREF	−0.0143136 ± 0.0251569	0.57	−0.0225991 ± 0.0411359	0.58	−0.0044129 ± 0.0331399	0.89	−0.034293 ± 0.0288664	0.24	0.0413532 ± 0.0533637	0.44
INTMED	−0.0035764 ± 0.0062928	0.57	−0.0055287 ± 0.0100953	0.58	−0.0011159 ± 0.0083809	0.89	−0.0133413 ± 0.0112737	0.24	0.0045682 ± 0.0059915	0.45
PIE	0.0243976 ± 0.0155735	0.12	0.0261129 ± 0.0241334	0.28	0.0222967 ± 0.0206908	0.28	0.0393186 ± 0.0246738	0.11	0.0022808 ± 0.0174407	0.90
LIN DNAmage										
TE	0.0161015 ± 0.1058051	0.88	−0.0319931 ± 0.1582704	0.84	0.0588192 ± 0.1418196	0.68	0.2113883 ± 0.1459292	0.15	−0.1942426 ± 0.14584	0.18
CDE	−0.0155356 ± 0.12573	0.90	0.023613 ± 0.1867545	0.90	−0.0127457 ± 0.170582	0.94	0.2303516 ± 0.169564	0.17	*−0.325804 ± 0.184643*	*0.078*
INTREF	−0.0031558 ± 0.0431648	0.94	−0.043412 ± 0.0676271	0.52	0.005537 ± 0.058362	0.92	−0.0625261 ± 0.0499875	0.21	0.1132211 ± 0.0894854	0.21
INTMED	−0.0007885 ± 0.0107853	0.94	−0.0106205 ± 0.0166157	0.52	0.0014001 ± 0.0147587	0.92	−0.0243253 ± 0.019531	0.21	0.012506 ± 0.01031	0.23
PIE	0.0355814 ± 0.0266483	0.18	−0.0015737 ± 0.0390555	0.97	*0.0646278 ± 0.0369201*	*0.080*	0.0678881 ± 0.0427146	0.11	0.0058338 ± 0.0292008	0.84
WEIDNER DNAmage										
TE	−0.0557187 ± 0.1498036	0.71	0.107925 ± 0.2263041	0.63	−0.1679082 ± 0.199936	0.40	−0.0622503 ± 0.2060429	0.76	0.0070123 ± 0.209054	0.97
CDE	−0.1272193 ± 0.1780053	0.48	0.1515346 ± 0.2664227	0.57	−0.2998148 ± 0.2407388	0.21	−0.0788371 ± 0.2394521	0.74	−0.1554603 ± 0.2646827	0.56
INTREF	0.014877 ± 0.0611136	0.81	−0.1053808 ± 0.096669	0.28	0.0758886 ± 0.0824655	0.36	−0.0539771 ± 0.0704672	0.44	0.1195995 ± 0.1280105	0.35
INTMED	0.0037172 ± 0.015273	0.81	−0.0257807 ± 0.0239415	0.28	0.01919 ± 0.0209451	0.36	−0.0209991 ± 0.0274587	0.44	0.0132126 ± 0.0144783	0.36
PIE	0.0529064 ± 0.0377582	0.16	0.0875519 ± 0.0574783	0.13	0.0368279 ± 0.0512003	0.472	0.0915628 ± 0.0602564	0.13	0.0296604 ± 0.0424281	0.49
VIDAL−BRALO DNAmage										

TE	0.1084912 ± 0.0702483	0.12	0.0178189 ± 0.1069319	0.89	0.1853357 ± 0.0933298	0.047	*0.18725 ± 0.0968462*	*0.053*	0.0378987 ± 0.0973401	0.70
CDE	0.0825509 ± 0.0834268	0.32	0.0592193 ± 0.1257899	0.64	0.135604 ± 0.1122966	0.23	*0.1932932 ± 0.1124779*	*0.086*	−0.0431816 ± 0.1231865	0.73
INTREF	−0.0071078 ± 0.028643	0.80	−0.0696407 ± 0.0457747	0.13	0.010139 ± 0.038424	0.79	−0.0439231 ± 0.0331697	0.19	0.0554439 ± 0.0596059	0.35
INTMED	−0.0017759 ± 0.0071583	0.80	−0.0170371 ± 0.0114663	0.14	0.0025639 ± 0.0097198	0.79	−0.0170879 ± 0.0129667	0.19	0.0061243 ± 0.006739	0.36
PIE	*0.034824 ± 0.0178459*	*0.051*	*0.0452775 ± 0.0273013*	*0.097*	0.0370288 ± 0.0241836	0.13	*0.0549677 ± 0.0284892*	*0.054*	0.0195121 ± 0.0200322	0.33
YANG DNAmage										
TE	−0.0002345 ± 0.000254	0.36	−0.000297 ± 0.0004213	0.48	−0.000184 ± 0.0003155	0.56	−0.0005553 ± 0.0003447	0.11	0.0002999 ± 0.0003676	0.42
CDE	−0.0004507 ± 0.0003019	0.14	−0.0004215 ± 0.0004968	0.40	−0.0004414 ± 0.0003799	0.25	−0.000656 ± 0.0004007	0.10	−0.0001649 ± 0.0004649	0.72
INTREF	0.0001327 ± 0.0001038	0.20	0.0000177 ± 0.0001797	0.92	0.0001863 ± 0.0001304	0.15	−0.0000283 ± 0.0001178	0.81	*0.0004184 ± 0.0002262*	*0.064*
INTMED	0.0000332 ± 0.0000261	0.20	0.0000043 ± 0.000044	0.92	0.0000471 ± 0.0000333	0.16	−0.000011 ± 0.0000458	0.81	*0.0000462 ± 0.0000272*	*0.09*
PIE	0.0000503 ± 0.0000636	0.43	0.0001025 ± 0.0001052	0.33	0.0000241 ± 0.0000806	0.76	0.00014 ± 0.0001007	0.16	0.0000002 ± 0.0000734	1.00
ZHANG DNAmage										
TE	0.0091244 ± 0.0059053	0.12	*0.0151187 ± 0.0090044*	*0.093*	0.0048389 ± 0.007841	0.54	*0.0152963 ± 0.0080487*	*0.057*	0.0016223 ± 0.00847	0.85
CDE	0.0051013 ± 0.0069908	0.47	0.0155272 ± 0.0105257	0.14	−0.001852 ± 0.0094171	0.84	0.0102421 ± 0.0093155	0.27	−0.0025159 ± 0.0107079	0.81
INTREF	−0.0013697 ± 0.0024007	0.57	−0.006127 ± 0.0038327	0.11	0.0012154 ± 0.0032226	0.71	−0.0025158 ± 0.0027429	0.36	0.0015634 ± 0.0051722	0.76
INTMED	−0.0003422 ± 0.0006005	0.57	−0.0014989 ± 0.0009624	0.12	0.0003073 ± 0.0008155	0.71	−0.0009787 ± 0.0010696	0.36	0.0001727 ± 0.0005728	0.76
PIE	0.0057351 ± 0.0015661	<0.001	0.0072175 ± 0.0024903	0.004	0.0051682 ± 0.0020837	0.013	0.0085487 ± 0.0024559	<0.001	0.0024021 ± 0.0017901	0.18
BOCKLANDT DNAmage										
TE	0.0008678 ± 0.0009813	0.38	0.0023248 ± 0.0015099	0.12	−0.0003879 ± 0.0012946	0.764	−0.0013589 ± 0.0012857	0.291	0.0035284 ± 0.0015488	0.023
CDE	0.0008572 ± 0.0011666	0.46	0.0015727 ± 0.0017754	0.38	−0.0002488 ± 0.0015592	0.873	−0.0015788 ± 0.0014949	0.291	0.0041047 ± 0.0019613	0.036
INTREF	0.0001334 ± 0.0004005	0.74	*0.0011334 ± 0.0006473*	*0.080*	−0.0001227 ± 0.0005335	0.818	0.0004192 ± 0.0004402	0.341	−0.0006225 ± 0.000948	0.511
INTMED	0.0000333 ± 0.0001001	0.74	*0.0002773 ± 0.0001634*	*0.090*	−0.000031 ± 0.0001349	0.818	0.0001631 ± 0.0001717	0.342	−0.0000688 ± 0.000106	0.516
PIE	−0.0001562 ± 0.0002457	0.53	*−0.000659 ± 0.000386*	*0.088*	0.0000146 ± 0.0003305	0.965	−0.0003624 ± 0.0003739	0.332	0.0001149 ± 0.0003111	0.712
GARAGNANI DNAmage										
TE	0.0004467 ± 0.0007591	0.56	0.0011673 ± 0.0011508	0.31	−0.0000577 ± 0.0010108	0.95	0.0016044 ± 0.0010513	0.13	−0.0007429 ± 0.0010294	0.47
CDE	0.0000815 ± 0.0009021	0.93	0.0012448 ± 0.0013568	0.36	−0.0006705 ± 0.001217	0.58	0.0017309 ± 0.0012225	0.16	−0.0018332 ± 0.0013022	0.16
INTREF	0.0001044 ± 0.0003097	0.74	−0.0003011 ± 0.0004913	0.54	0.000324 ± 0.0004167	0.44	−0.0003064 ± 0.0003599	0.40	0.0007811 ± 0.0006309	0.22
INTMED	0.0000261 ± 0.0000774	0.74	−0.0000737 ± 0.0001207	0.54	0.0000819 ± 0.0001057	0.44	−0.0001192 ± 0.0001403	0.40	0.0000863 ± 0.0000726	0.24
PIE	0.0002346 ± 0.000191	0.22	0.0002973 ± 0.0002878	0.30	0.0002069 ± 0.000259	0.43	0.0002991 ± 0.0003058	0.33	0.0002229 ± 0.0002127	0.30
DNAm GRIMAGE										
TE	0.1761732 ± 0.0612903	0.004	0.020766 ± 0.0956298	0.83	0.2956451 ± 0.0799387	<0.001	0.1952763 ± 0.0820687	0.017	0.1509621 ± 0.0919291	0.10
CDE	0.1136006 ± 0.0724519	0.12	−0.0636511 ± 0.1122953	0.57	0.2564346 ± 0.095593	0.007	0.1048024 ± 0.0948908	0.27	0.1208863 ± 0.1159003	0.30
INTREF	−0.0053663 ± 0.0248744	0.83	0.0205033 ± 0.0406476	0.61	−0.0328315 ± 0.0327533	0.32	−0.0038113 ± 0.0279016	0.89	−0.0061629 ± 0.0559677	0.91
INTMED	−0.0013408 ± 0.0062161	0.83	0.005016 ± 0.0099706	0.62	−0.0083021 ± 0.0083257	0.32	−0.0014828 ± 0.0108555	0.89	−0.0006811 ± 0.0061844	0.91
PIE	0.0692797 ± 0.0165738	<0.001	0.0588978 ± 0.0253337	0.020	0.0803441 ± 0.0222938	<0.001	0.095768 ± 0.0252964	<0.001	*0.0369198 ± 0.0204061*	*0.070*
MPOA										
TE	0.0032687 ± 0.0012509	0.009	0.0019199 ± 0.001945	0.32	0.0042659 ± 0.0016386	0.009	0.0036355 ± 0.0016903	0.031	0.0027659 ± 0.0018405	0.13
CDE	0.0031488 ± 0.001482	0.034	0.0011998 ± 0.0022912	0.60	0.0045412 ± 0.0019641	0.021	*0.0032516 ± 0.0019614*	*0.097*	0.0033033 ± 0.0023126	0.15
INTREF	−0.000746 ± 0.0005098	0.14	−0.0000056 ± 0.0008288	1.00	*−0.001229 ± 0.000675*	*0.069*	−0.0006144 ± 0.0005778	0.29	−0.0012455 ± 0.0011197	0.27
INTMED	−0.0001864 ± 0.0001283	0.15	−0.0000014 ± 0.0002028	1.00	*−0.000311 ± 0.000174*	*0.073*	−0.000239 ± 0.0002255	0.29	−0.0001376 ± 0.0001278	0.28
PIE	0.0010524 ± 0.000327	0.001	0.000727 ± 0.0004933	0.14	0.001265 ± 0.0004413	0.004	0.0012373 ± 0.0005022	0.014	0.0008456 ± 0.0004196	0.044

Abbreviations: CDE: Controlled Direct Effect; CES−D: Centers for Epidemiological Studies−Depression; DNAm: DNA methylation; DNAmage: DNA methylation age; INTMED: Mediated Interaction; INTREF: Interaction referent; PIE: Pure Indirect Effect; TE: Total Effect. [a]OLS regression models with epigenetic clocks as alternative outcomes and point RPD measured in 2010−2012 as exposures and CES−D scores measured in 2014−2016 as a potential mediator, sample size N = 2,806, four−way mediation analysis. Stratified analysis by sex and/or race was also presented. Italicized findings have p < 0.10 but >0.05.
[b]Exogenous variables are the ones included in Table 2, Model 1, as covariates. See Covariates section for detail.

Supplementary Table 5. Perceived discrimination (EOD/RPD: 2010/2012) → depressive symptoms (CES−D: 2014/2016) → epigenetic clocks (2016): Structural Equations Modeling, overall, reduced model: HRS 2010-2016[a,b].

	EOD					RPD				
	Total effect	EOD→CESD→EPICLOCK Indirect effect	EOD →EPICLOCK Direct effect α13	EOD →CESD α12	CESD →EPICLOCK α23	Total effect	RPD→CESD→ EPICLOCK Indirect effect	RPD →EPICLOCK Direct effect α13	RPD →CESD α12	CESD →EPICLOCK α23
HORVATH DNAmage	−0.0062 ± 0.029	−0.0002 ± 0.0071	−0.0060 ± 0.030	+0.106 ± 0.008	−0.002 ± 0.067	−0.0244 ± 0.084	−0.0003 ± 0.0167	−0.024 ± 0.085	+0.251 ± 0.024	−0.001 ± 0.067
	P = 0.833	*P* = 0.98	*P* = 0.84	*P* < 0.001	*P* = 0.98	*P* = 0.77	*P* = 0.99	*P* = 0.78	*P* < 0.001	*P* = 0.78
HANNUM DNAmage	+0.0013 ± 0.0236	+0.016 ± 0.006	−0.015 ± 0.024	+0.106 ± 0.008	+0.156 ± 0.054	+0.0009 ± 0.0671	+0.0387 ± 0.0139	−0.0379 ± 0.0683	+0.251 ± 0.023	+0.154 ± 0.055
	P = 0.96	*P* = 0.005	*P* = 0.53	*P* < 0.001	*P* = 0.004	*P* = 0.99	*P* = 0.005	*P* = 0.58	*P* < 0.001	*P* < 0.001
LEVINE DNAmage	−0.0030 ± 0.0313	+0.032 ± 0.008	−0.035 ± 0.032	+0.106 ± 0.008	+0.1564 ± 0.054	+0.041 ± 0.088	+0.073 ± 0.019	−0.032 ± 0.090	+0.251 ± 0.024	+0.292 ± 0.071
	P = 0.92	P < 0.001	*P* = 0.27	*P* < 0.001	*P* = 0.004	*P* = 0.64	*P* < 0.001	*P* = 0.72	*P* < 0.001	*P* < 0.001
HORVATH 2 DNAmage	+0.025 ± 0.020	+0.008 ± 0.0055	+0.018 ± 0.021	+0.105 ± 0.008	+0.073 ± 0.046	+0.080 ± 0.057	+0.018 ± 0.012	+0.062 ± 0.058	+0.251 ± 0.024	+0.073 ± 0.046
	P = 0.20	*P* = 0.12	*P* = 0.39	*P* < 0.001	*P* = 0.11	*P* = 0.16	*P* = 0.12	*P* = 0.29	*P* < 0.001	*P* = 0.11
LIN DNAmage	+0.015 ± 0.034	*+0.014 ± 0.008*	+0.002 ± 0.035	+0.106 ± 0.008	*+0.132 ± 0.079*	+0.011 ± 0.098	*+0.034 ± 0.020*	−0.024 ± 0.100	+0.251 ± 0.024	*+0.137 ± 0.078*
	P = 0.65	*P = 0.097*	*P* = 0.96	*P* < 0.001	*P = 0.094*	*P* = 0.91	*P = 0.085*	*P* = 0.81	*P* < 0.001	*P = 0.080*
WEIDNER DNAmage	−0.030 ± 0.049	+0.027 ± 0.012	−0.056 ± 0.050	+0.106 ± 0.008	+0.253 ± 0.050	−0.025 ± 0.140	+0.059 ± 0.028	−0.085 ± 0.141	+0.251 ± 0.023	+0.237 ± 0.111
	P = 0.55	*P* = 0.026	*P* = 0.26	*P* < 0.001	*P* = 0.023	*P* = 0.86	*P* = 0.036	*P* = 0.55	*P* < 0.001	*P* = 0.032
VIDAL−BRALO DNAmage	−0.003 ± 0.023	+0.016 ± 0.006	−0.018 ± 0.024	+0.106 ± 0.008	+0.147 ± 0.052	+0.101 ± 0.064	+0.032 ± 0.013	+0.069 ± 0.066	+0.251 ± 0.023	+0.127 ± 0.052
	P = 0.91	*P* = 0.006	*P* = 0.44	*P* < 0.001	*P* = 0.005	*P* = 0.12	*P* = 0.017	*P* = 0.30	*P* < 0.001	*P* = 0.014
YANG DNAmage	+0.0000 ± 0.0001	+0.0023 ± 0.0005	−0.0000 ± 0.0001	+0.106 ± 0.008	+0.0004 ± 0.0002	−0.0001 ± 0.0002	+0.00011 ± 0.00005	−0.0002 ± 0.0002	+0.251 ± 0.024	+0.0004 ± 0.0002
	P = 1.00	P < 0.001	*P* = 0.61	*P* < 0.001	*P* = 0.030	*P* = 0.67	*P* = 0.028	*P* = 0.39	*P* < 0.001	*P* = 0.025
ZHANG DNAmage	+0.0008 ± 0.0019	+0.0023 ± 0.0005	−0.002 ± 0.002	+0.106 ± 0.008	+0.022 ± 0.004	+0.008 ± 0.005	+0.0052 ± 0.0012	−0.0029 ± 0.0055	+0.251 ± 0.024	+0.021 ± 0.004
	P = 0.68	P < 0.001	*P* = 0.44	*P* < 0.001	*P* < 0.001	*P* = 0.14	*P* < 0.001	*P* = 0.60	*P* < 0.001	*P* < 0.001
BOCKLANDT DNAmage	+0.0007 ± 0.0003	+0.0008 ± 0.0004	−0.0001 ± 0.0001	+0.106 ± 0.008	−0.0006 ± 0.0007	+0.0010 ± 0.0009	−0.0001 ± 0.0002	+0.0011 ± 0.0009	+0.251 ± 0.024	−0.0004 ± 0.0007
	P = 0.027	*P* = 0.019	*P* = 0.39	*P* < 0.001	*P* = 0.39	*P* = 0.27	*P* = 0.58	*P* = 0.23	*P* < 0.001	*P* = 0.58
GARAGNANI DNAmage	+0.00035 ± 0.00025	+0.0002 ± 0.0003	+0.0002 ± 0.0003	*+0.00011 ± 0.00006*	*+0.0010 ± 0.00057*	+0.0005 ± 0.0007	*+0.0003 ± 0.0001*	+0.0002 ± 0.0007	+0.251 ± 0.024	*+0.0011 ± 0.0006*
	P = 0.15	*P* = 0.33	*P* = 0.33	*P = 0.079*	*P = 0.076*	*P* = 0.49	*P = 0.05*	*P* = 0.77	*P* < 0.001	*P = 0.05*
DNAm GRIMAGE	+0.0254 ± 0.020	+0.030 ± 0.005	−0.0048 ± 0.0204	+0.1057 ± 0.0083	+0.286 ± 0.045	+0.170 ± 0.57	+0.067 ± 0.013	*+0.1028 ± 0.0574*	+0.251 ± 0.024	+0.268 ± 0.045
	P = 0.20	P < 0.001	*P* = 0.82	*P* < 0.001	*P* < 0.001	*P* = 0.003	P < 0.001	*P = 0.073*	*P* < 0.001	*P* < 0.001
MPOA	+0.00033 ± 0.00041	+0.00035 ± 0.00010	−0.0000 ± 0.0004	+0.1057 ± 0.0083	+0.0033 ± 0.0009	+0.0026 ± 0.0011	+0.00074 ± 0.00024	+0.0018 ± 0.0012	+0.251 ± 0.024	+0.0030 ± 0.0009
	P = 0.41	*P* = 0.001	*P* = 0.98	*P* < 0.001	*P* < 0.001	*P* = 0.024	*P* = 0.002	*P* = 0.12	*P* < 0.001	*P* = 0.001

Abbreviations: CES−D: Centers for Epidemiological Studies−Depression; DNAm: DNA methylation; DNAmage: DNA methylation age; EOD: Experience of Discrimination; HRS: Health and Retirement Study; RPD: Reasons for Perceived Discrimination; SEM: Structural Equations Modeling. [a]Structural Equations Models with Y = EPICLOCK at v_3, X = Perceived discrimination scores (EOD/RPD) at v0v1 and mediator M (CES−D total score) at v2v3. Italicized findings have $p < 0.10$ but >0.05. [b]Exogenous variables were age in 2016, sex and race/ethnicity.
